# Bond Softening and Elastic Confinement of Monolithic Dense Binary Alloys for High Volumetric Capacity of K‐Ion Battery Anodes

**DOI:** 10.1002/anie.9686588

**Published:** 2026-03-23

**Authors:** Yunyong Li, Yiru Zhou, Bingchun Wang, Xinying Wang, Lei Liu, Zhuhang Shao, Hao Wu, Zaowen Zhao, Yingqiang Wu, Xuerong Zheng, Xinwei Li, Wenwu Li, Yida Deng, Zaiping Guo, Ho Seok Park

**Affiliations:** ^1^ State Key Laboratory of Tropic Ocean Engineering Materials and Materials Evaluation School of Materials Science and Engineering Hainan University Haikou P.R. China; ^2^ School of Materials and Energy Guangdong University of Technology Guangzhou P.R. China; ^3^ School of Chemical Engineering Sungkyunkwan University Suwon South Korea; ^4^ School of Chemical Engineering The University of Adelaide Adelaide Australia; ^5^ SKKU Institute of Energy Science and Technology (SIEST) Sungkyunkwan University (SKKU) Suwon Gyeonggi‐do Republic of Korea

**Keywords:** bond softening, dense encapsulation structure, elastic confinement, potassium‐ion batteries, two‐dimensional alloy anode

## Abstract

Two‐dimensional (2D) Sb─Bi alloys are considered as the promising high‐capacity and high‐rate anodes of potassium‐ion batteries, yet their practical application is limited by low density and structural degradation. Herein, we propose an intrinsic‐extrinsic dual‐stabilization strategy that integrates 2D binary Sb_0.6_Bi_0.4_ nanosheets into 3D elastic graphene networks, constructing a highly dense monolithic architecture (HD‐Sb_0.6_Bi_0.4_@G). This design features with high density (2.6 g cm^−3^) and electrical conductivity (555.6 S m^−1^), delivering large volumetric capacity (1355.1 mAh cm^−3^) and high areal capacity (11.4 mAh cm^−2^) at an ultra‐high loading of 27.6 mg cm^−2^, along with long‐term cyclability (65.4% capacity retention after 1500 cycles) and stable full‐cell performance. Experimental and theoretical analyses reveal that the Sb─Bi alloy exhibited “bond softening” with the optimized interlayer spacing and moderate bond energy, facilitating rapid K^+^ diffusion and buffering strain. Furthermore, the elastic graphene network provides nanoscale confinement, accommodating volume expansion and preserving structural and electrical integrity. Strong electronic coupling at the alloy‐graphene interface further reduces K^+^ adsorption and diffusion energy barriers, enabling fast ion transport under the high‐loading anodes. This intrinsic‐extrinsic synergy between binary‐alloy bond softening and nanoscale elastic confinement provides a universal strategy for compact, high‐loading electrodes with high volumetric and areal energy storage.

## Graphical abstract

Synergistically intrinsic‐extrinsic dual‐stabilization engineering to construct dense monolithic alloy architectures is proposed by integrating 2D binary alloy nanosheets into 3D elastic graphene networks. Such a dense monolithic anode achieves high volumetric and areal performance, enabling fast K^+^ transfer and superior structural recovery during potassiation/depotassiation.

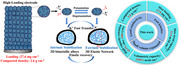

## 1 Introduction

The growing demand for compact and high‐performance energy storage systems in portable electronics and electric vehicles has accelerated the search for rechargeable batteries combining high gravimetric and volumetric energy densities [1, 2, 3]. Although lithium‐ion batteries (LIBs) remain commercially dominant, their progress is increasingly constrained by limited volumetric capacity, excessive inactive components, rising resource costs, and safety concerns [4, 5]. Potassium‐ion batteries (PIBs) have emerged as a competitive alternative to LIBs, benefiting from similar electrochemical mechanisms and the lower cost of potassium [6]. Nevertheless, the large K^+^ ionic radius (∼1.38 Å) results in sluggish kinetics, high diffusion barriers, and severe volume expansion in most anode materials such as phosphides [7, 8, 9, 10], and metal chalcogenides [11, 12, 13], thereby leading to rapid structural degradation and limited cycling stability, particularly under high‐loading thick electrode conditions to enhance areal capacity [14].

Alloy‐type anodes [15], especially Sb‐ and Bi‐based materials, are among the most attractive candidates for PIBs owing to their high theoretical capacities and favorable alloying potentials. Bismuth exhibits excellent electrical conductivity (7.75 × 10^5^ S m^−1^) and low reaction hysteresis but suffers from relatively low capacity (386 mAh g^−1^) [16, 17, 18, 19, 20]. Antimony, by contrast, features high theoretical capacity (660 mAh g^−1^) and suitable redox properties but undergoes drastic volume fluctuations (∼400%) during potassiation, causing fracture, pulverization, and unstable solid‐electrolyte interfaces [21, 22, 23]. Alloying Sb and Bi at the atomic scale can synergistically tailor interlayer spacing, electronic structure, and mechanical response, yielding improved ion mobility and structural tolerance beyond single‐metal systems [12, 24, 25]. In particular, 2D binary alloys (e.g., Sb─Bi) could theoretically optimize interlayer spacing and bond energy, provide tunable electronic behavior and more K^+^ storage sites (large surface area), enabling improved K‐storage kinetics and robust structural reversibility [26]. However, their inherently low tap density, interfacial stress accumulation, and insufficient structural integrity under high‐loading thick electrode conditions remain critical barriers to achieving practical high‐volumetric‐energy PIBs [27, 28, 29, 30].

Although carbon compositing and nanoengineering strategies can partially buffer alloy volume changes and improve conductivity, conventional carbon frameworks are often mechanically insufficient for suppressing nanoscale strain or are too porous to enable compact electrode architectures, resulting in limited volumetric performance and inferior long‐term integrity [31, 32, 33, 34, 35]. Thus, a major challenge lies in simultaneously optimizing three interdependent factors: (1) intrinsic K^+^ diffusion kinetics and structural resilience of the 2D binary alloy phase; (2) robust macroscopic electrode densification without sacrificing ion/electron accessibility; and (3) stable mechanical/electronic integrity under high‐loading thick electrode conditions. Consequently, constructing compact thick electrodes from 2D binary alloys that simultaneously leverage their intrinsic advantages and mitigate key drawbacks is pivotal for realizing high volumetric and areal performance.

**FIGURE 1:**
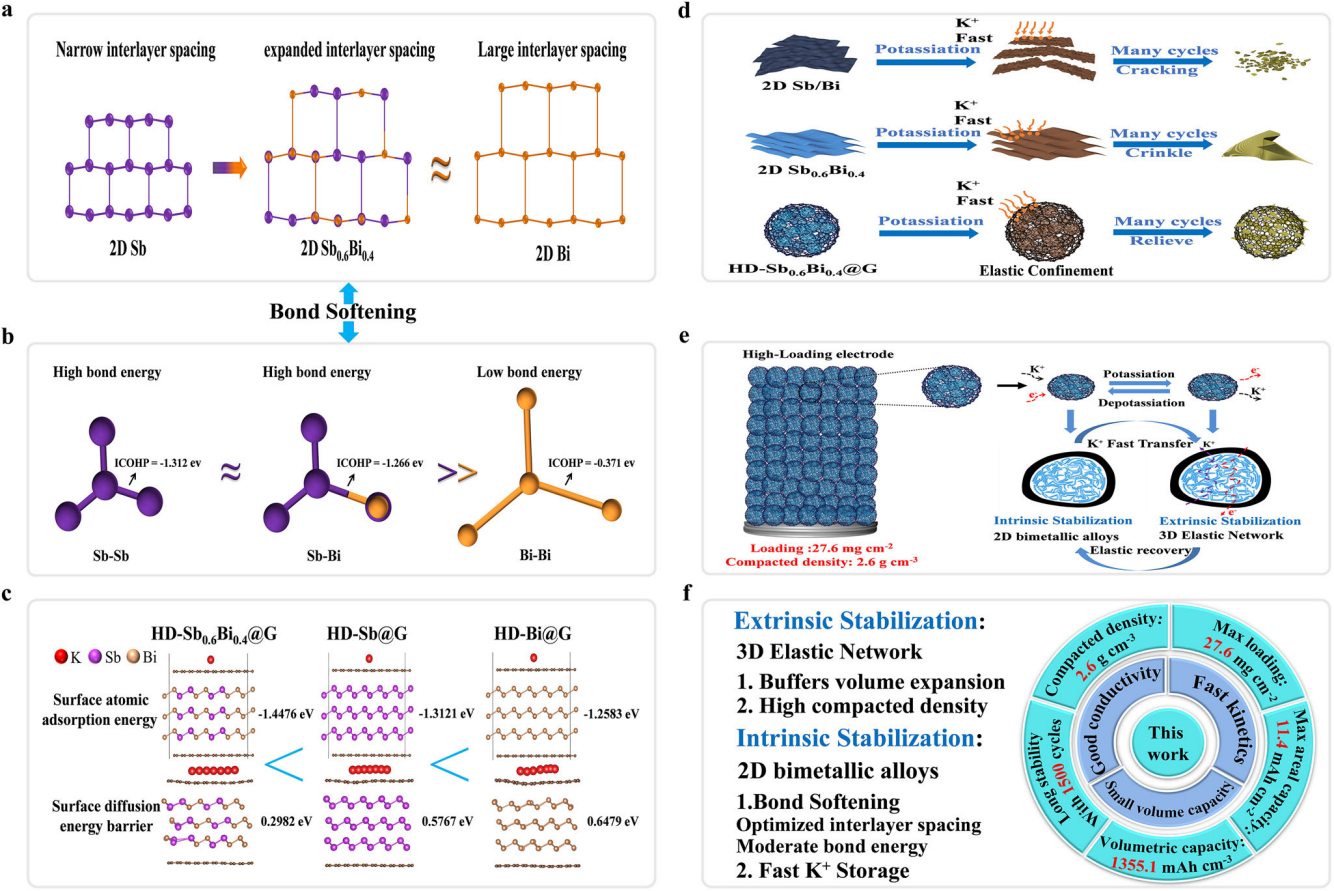
Structural and mechanistic advantages of 2D Sb_0.6_Bi_0.4_ and HD‐Sb_0.6_Bi_0.4_@G for potassium storage. (a) Comparison of interlayer spacing in Sb, Bi, and Sb_0.6_Bi_0.4_ alloy. (b) Bond energy comparison illustrating enhanced alloy stability. (c) Calculated K^+^ adsorption energies and diffusion energy barriers on HD‐Sb_0.6_Bi_0.4_@G. (d) High structural robustness of high‐density HD‐Sb_0.6_Bi_0.4_@G monolith. (e) Schematic illustration of the structural evolution during the potassiation/depotassiation process. (f) Representative electrochemical performance metrics demonstrating superior K‐storage capability.

Herein, we propose a synergistically intrinsic‐extrinsic dual‐stabilization strategy to construct a highly dense monolithic anode (HD‐Sb_0.6_Bi_0.4_@G) by integrating 2D Sb_0.6_Bi_0.4_ nanosheets into a 3D elastic graphene network. Intrinsically, density functional theory (DFT) calculations confirm that the binary alloy exhibits a “bond‐softening” effect: its interlayer spacing expands close to that of Bi (Figure 1a), while the Sb─Bi bond energy (integrated crystal orbital Hamiltonian population [ICOHP] = −1.266 eV, Figure 1b) resides between those of Sb─Sb and Bi─Bi bonds [36, 37]. This moderate bond strength, coupled with an optimized interlayer structure, collectively lowers K^+^ adsorption energy and diffusion barrier, thereby enhancing ion‐transport kinetics and structural flexibility (Figure 1c). Extrinsically, the elastic graphene network uniformly encapsulates the 2D alloy nanosheets, providing nanoscale confinement that buffers volume fluctuations, maintains mechanical/electrical continuity, and ensures long‐term integrity of the dense electrode (Figure 1d,e). Benefiting from this coupled stabilization, the HD‐Sb_0.6_Bi_0.4_@G architecture breaks the traditional trade‐off between kinetics, stability, and packing density in alloy‐based PIB anodes, enabling high volumetric/areal capacities and remarkable long‐term cycling stability (Figure 1f).

## 2 Results and Discussion

### 2.1 Architecture Construction, Microstructure, and Multiscale Characterization of the HD‐Sb_0.6_Bi_0.4_@G Monolith

Figure S1 schematically illustrates the fabrication of the high‐density HD‐Sb_0.6_Bi_0.4_@G monolith, which proceeds through four key steps: (1) 2D Sb_0.6_Bi_0.4_ nanosheets were synthesized via a one‐step co‐deposition reaction between Sb^3+^, Bi^3+^, and Fe in an acidic medium (Fe + 0.6 Sb^3+^ + 0.4 Bi^3+^ → Sb_0.6_Bi_0.4_ + Fe^3+^); (2) A small amount of graphene oxide (GO, ∼15 wt%) is introduced as a structural‐directing agent to form a freestanding Sb_0.6_Bi_0.4_@reduced graphene oxide (Sb_0.6_Bi_0.4_@RGO) hydrogel through a solvothermal assembly at 200°C; (3) Ambient drying induces hydrogel self‐shrinkage, forming a densely packed Sb_0.6_Bi_0.4_@RGO monolith; (4) Microwave‐assisted annealing under H_2_/Ar atmosphere enhances electrical conductivity, removes residual oxygen‐containing groups, and promotes further densification, ultimately yielding the compact HD‐Sb_0.6_Bi_0.4_@G monolith.

**FIGURE 2:**
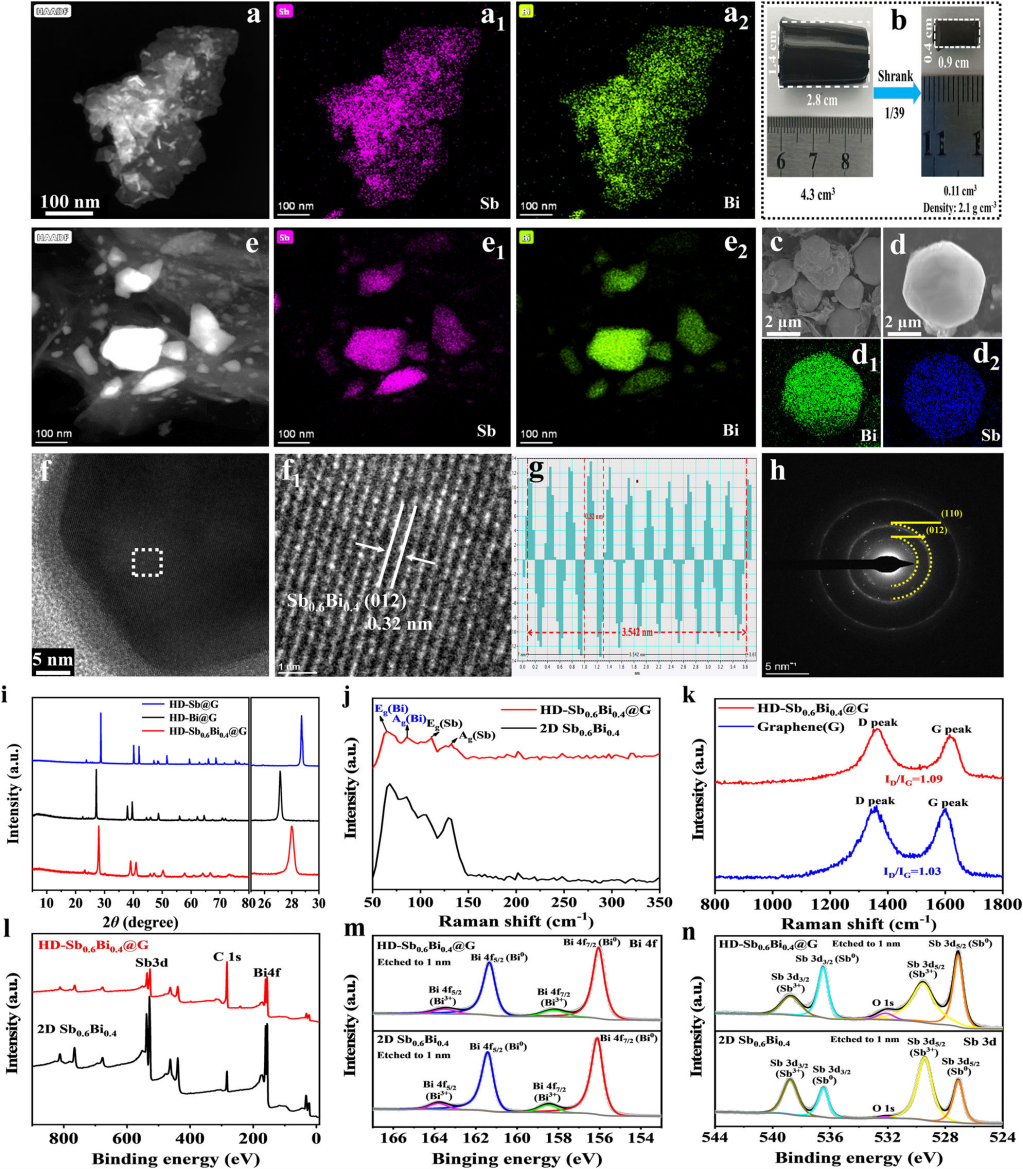
Morphology, microstructure, and phase characterization of dense HD‐Sb_0.6_Bi_0.4_@G. (a) HAADF‐STEM image of 2D Sb_0.6_Bi_0.4_ and elemental mapping of (a_1_) Sb and (a_2_) Bi. (b) Shrinkage of Sb_0.6_Bi_0.4_@RGO hydrogel into dense HD‐Sb_0.6_Bi_0.4_@G monolith. (c) SEM image of HD‐Sb_0.6_Bi_0.4_@G particles and (d) elemental mapping of (d_1_) Bi, (d_2_) Sb. (e) HAADF‐STEM image and elemental mapping of (e_1_) Sb and (e_2_) Bi. (f,f1) HR‐TEM images. (g) Fourier transform analysis. (h) SAED pattern. (i) XRD patterns: HD‐Sb@G, HD‐Bi@G, and HD‐Sb_0.6_Bi_0.4_@G. (j) Raman spectra: HD‐Sb_0.6_Bi_0.4_@G and 2D Sb_0.6_Bi_0.4_. (k) Comparative Raman spectra: HD‐Sb_0.6_Bi_0.4_@G and graphene. (l) XPS survey spectra: HD‐Sb_0.6_Bi_0.4_@G and 2D Sb_0.6_Bi_0.4_. (m) High‐resolution Bi 4f XPS spectrum. (n) High‐resolution Sb 3d XPS spectrum.

The microstructure and morphology of HD‐Sb_0.6_Bi_0.4_@G were investigated using scanning electron microscopy (SEM) and transmission electron microscopy (TEM). As shown in Figures S2 and 2a–a2, the as‐prepared Sb_0.6_Bi_0.4_ displays a well‐dispersed 2D nanosheet architecture with homogeneous distribution of Sb and Bi, as confirmed by TEM‐EDS mapping. Following integration with graphene, a cylindrical hydrogel structure is formed (Figure 2b), which undergoes substantial volumetric contraction during drying, achieving a 39‐fold reduction in volume (final volume = 1/39 of the original). The resulting monolith displays a high density of 2.1 g cm^−3^ (calculation details in Method S1). Tap density measurements further validate the compact architecture. After 1000 taps, Sb_0.6_Bi_0.4_ exhibits a loose density of 0.67 g cm^−3^, whereas HD‐Sb_0.6_Bi_0.4_@G reaches 1.93 g cm^−3^ (Figure S3), emphasizing the significantly enhanced packing density derived from the monolithic structure.

TEM imaging (Figure S4) confirms the conformal encapsulation of Sb_0.6_Bi_0.4_ nanosheets by thin graphene layers, preserving their 2D morphology during solvothermal assembly. SEM imaging (Figure 2c) reveals densely packed microspheres, and EDS elemental mapping (Figure 2d–d2,e–e2) demonstrates uniform distribution of Sb and Bi throughout the composite. High‐resolution TEM (Figure 2f,g) shows clear lattice fringes with a spacing of 0.32 nm, corresponding to the (012) plane (PDF#35‐0517), while the selected‐area electron diffraction (SAED) pattern (Figure 2h) displays diffraction rings indexed to the (012) and (110) planes of the Sb_0.6_Bi_0.4_ alloy, confirming its high crystallinity and alloy phase purity. These results collectively demonstrate the successful synthesis of homogeneous 2D Sb_0.6_Bi_0.4_, with a 3D dense graphene encapsulation structure constructed on the 2D Sb_0.6_Bi_0.4_ substrate.

X‐Ray diffraction (XRD) analysis confirms the crystalline phase of both HD‐Sb_0.6_Bi_0.4_@G and the pristine 2D alloy, with sharp diffraction peaks indicative of high crystallinity, and a pattern consistent with the SbBi reference (PDF #35‐0517) (Figure S5A) [38], confirming the successful synthesis of the materials. A comparative XRD pattern analysis of HD‐Sb_0.6_Bi_0.4_@G, HD‐Sb@G, and HD‐Bi@G (Figure 2i) shows that the d‐spacing of the (012) plane in the Sb_0.6_Bi_0.4_ alloy is 0.318 nm, intermediate between pure Sb (0.310 nm) and Bi (0.328 nm). This lattice expansion relative to Sb (+2.6%) corresponds to a left shift in the diffraction angle (Δ2*θ* = −0.7°).

Raman spectroscopy (Figure 2j) further verifies the phase composition, displaying characteristic *E*_g_ and *A*_g_ modes of Bi (67.9, 85.5 cm^−1^) and Sb (105.6, 129.3 cm^−1^) without impurity peaks [32, 39]. Figure 2k presents the local Raman spectra of HD‐Sb_0.6_Bi_0.4_@G and graphene processed under identical solvothermal and microwave‐thermal conditions. The *I_D_/I_G_* ratio of pristine graphene was measured to be 1. After compositing with 2D Sb_0.6_Bi_0.4_, the *I_D_/I_G_* of graphene increased to 1.09, suggesting the introduction of more defects [40, 41]. Current‐voltage (*I*‐*V*) analysis (Figure S5B) demonstrates a remarkable increase in electrical conductivity from 62 to 555.6 S m^−1^ after microwave‐assisted annealing, attributed to removal of oxygen‐containing groups and restoration of conjugated graphene networks.

X‐Ray photoelectron spectroscopy (XPS) was used to probe the surface chemical states of HD‐Sb_0.6_Bi_0.4_@G and 2D Sb_0.6_Bi_0.4_ after 1 nm surface etching (Figure 2l). In the high‐resolution Bi 4f spectra (Figure 2m), both materials show dominant Bi peaks with only weak Bi^3+^ signals, indicative of an ultrathin surface oxide layer [42, 43]. The Sb 3d spectra (Figure 2n) further reveal that the O 1s signal is notably weaker in 2D Sb_0.6_Bi_0.4_, confirming minimal oxidation. More importantly, while the Sb intensity in the 2D alloy is slightly lower than that of Sb^3+^, it becomes stronger than Sb^3+^ in HD‐Sb_0.6_Bi_0.4_@G. This clear enhancement of the metallic Sb state demonstrates that the reductive thermal treatment effectively removes surface oxygen from the alloy. The residual O 1s peak at 532.2 eV in the composite is attributed to oxygen‐containing groups on the graphene surface [30, 43].

In summary, these results confirm the successful synthesis of 2D Sb_0.6_Bi_0.4_ nanosheets and their transformation into a high‐density, highly conductive graphene‐encapsulated monolithic architecture with preserved 2D alloy integrity, optimized interfacial contact, and enhanced electronic conductivity.

### 2.2 Evaluation of the Electrochemical Properties of HD‐Sb_0.6_Bi_0.4_@G

**FIGURE 3:**
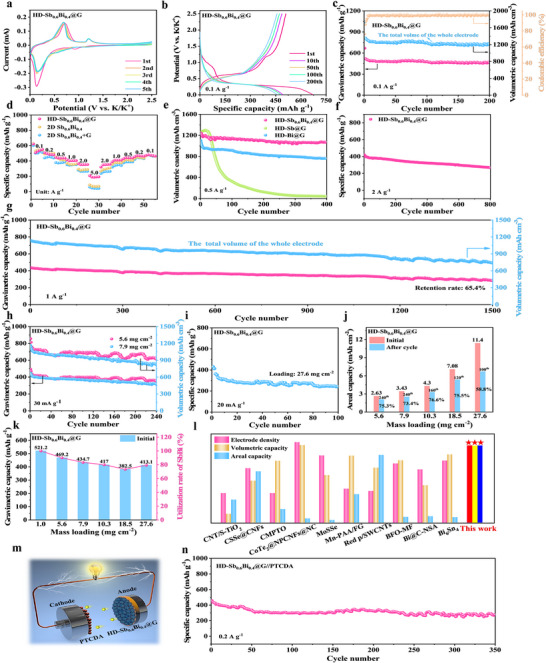
HD‐Sb_0.6_Bi_0.4_@G anode performance in PIBs. (a) First five cycles CV @ 0.1 mV s^−1^. (b) GCD curves @ 0.1 A g^−1^. (c) Cycling @ 0.1 A g^−1^. (d) Rate capability: composite versus 2D alloy versus mixture. (e) Cycling comparison: SbBi versus Sb versus Bi composites @ 0.5 A g^−1^. (f) High‐rate cycling (2 A g^−1^). (g) Long‐life cycling (1 A g^−1^). (h) Cycling under different mass loadings. (i) Stability @ 27.6 mg cm^−2^. (j) Areal capacity versus loading. (k) Utilization rate versus loading. (l) Benchmarking with other thick anodes. (m) Working mechanism. (n) Full‐cell (vs. PTCDA) cycling @ 0.2 A g^−1^.

The HD‐Sb_0.6_Bi_0.4_@G composite forms a dense monolithic architecture encapsulated within a conductive 3D graphene network, achieving a favorable synergy between Sb and Bi for high capacity and exceptional cycling stability in PIBs. Figure 3a presents the initial five CV cycles at 0.1 mV s^−1^. A faint reduction peak near 0.95 V corresponds to the initial alloying of the SbBi phase, while a broad, intense peak between 0.01 and 0.4 V arises from SEI formation and subsequent conversion to K_3_(SbBi) [44]. During anodic scans, reversible peaks at 0.7 and 1.2 V correspond to the stepwise dealloying of K_3_(SbBi) to SbBi [32]. The strong overlap of successive cycles indicates high electrochemical reversibility, further supported by similar CV features in control samples (Figure S6). GCD profiles at 0.1 A g^−1^ (Figure 3b) show initial discharge/charge capacities of 669 and 510.6 mAh g^−1^, with a first‐cycle Coulombic efficiency of 76.3%. As shown in Figure 3c, the electrode retains 459.6 mAh g^−1^ after 200 cycles, corresponding to 88.2% capacity retention relative to the second cycle. Rate performance comparisons among HD‐Sb_0.6_Bi_0.4_@G, 2D Sb_0.6_Bi_0.4_, and its graphene mixture highlight the structural advantage of the 3D graphene encapsulation. EIS analysis (Figure S7) reveals that HD‐Sb_0.6_Bi_0.4_@G exhibits the lowest ohmic resistance (6.96 Ω) and charge‐transfer resistance (420 Ω), confirming that the graphene network enhances electronic conduction, facilitates K^+^ diffusion, and mitigates volume change during cycling, thus contributing to superior rate capability and long‐term stability [45].

As shown in Figure 3d, the HD‐Sb_0.6_Bi_0.4_@G electrode delivers gravimetric capacities of 509.1, 487.5, 436.4, 402.8, 351.4, and 187.2 mAh g^−1^ at current densities from 0.1 to 5 A g^−1^. These values are significantly higher than those of the 2D Sb_0.6_Bi_0.4_ and 2D Sb_0.6_Bi_0.4_+G electrodes at each current density, highlighting its superior rate performance. This enhancement is attributed not only to the presence of graphene but also to the effective encapsulation of 2D Sb_0.6_Bi_0.4_ within the 3D graphene network, which creates a highly conductive and robust monolithic structure that facilitates electron transport and charge‐transfer kinetics.

Figure 3e compares the long‐term cycling performance of HD‐Sb_0.6_Bi_0.4_@G, HD‐Sb@G, and HD‐Bi@G electrodes at 0.5 A g^−1^. Initially, HD‐Sb@G shows the highest volumetric capacity but suffers from rapid decay, while HD‐Bi@G exhibits better cycling stability but a lower volumetric capacity due to its lower mass‐specific capacity. In contrast, HD‐Sb_0.6_Bi_0.4_@G achieves a balanced synergy between Sb and Bi, resulting in both high volumetric capacity and excellent cycling stability. It delivers a volumetric capacity of 1069.4 mAh cm^−3^ and retains 85.7% after 400 cycles.

The dense monolithic structure of HD‐Sb_0.6_Bi_0.4_@G leads to a high electrode compaction density of 2.6 mg cm^−3^ (Figure S8). Consequently, the electrode achieves a maximum volumetric capacity of 1355.1 mAh cm^−3^ at 0.1 A g^−1^, maintaining 1195 mAh cm^−3^ after 200 cycles Methods (S3). This demonstrates that the dense HD‐Sb_0.6_Bi_0.4_@G electrode successfully integrates high electrode density with ultrahigh volumetric capacity. The encapsulated architecture of HD‐Sb_0.6_Bi_0.4_@G, featuring an integrated conductive graphene network, facilitates rapid K^+^ diffusion and charge transfer. This, combined with the flexible matrix that accommodates volume changes of the Sb_0.6_Bi_0.4_ alloy and ensures structural integrity, results in excellent high‐rate, long‐term cycling stability.

At 2 A g^−1^ (Figure 3f), the electrode delivers a stable capacity of 269.4 mAh g^−1^ after 800 cycles (64% retention). At 1 A g^−1^ (Figure 3g), a mass‐specific capacity of 285.9 mAh g^−1^ (743.3 mAh cm^−3^) is maintained after 1500 cycles, with a high retention of 65.4% and an ultralow average capacity decay of only 0.023% per cycle. This performance stems from the synergy between Sb (contributing high capacity) and Bi (providing inherent stability), further enhanced by the 3D graphene encapsulation, which forms a dense, stable monolith with superior electron/ion transport. This compact structure effectively addresses the challenges of long‐distance electron conduction in thick electrodes.

Electrochemical performance under increasing mass loadings confirms this structural advantage (Figure 3h–k). With loadings of 5.6 and 7.9 mg cm^−2^ at 30 mA g^−1^, the electrodes maintain gravimetric capacities of 353.2 and 319 mAh g^−1^ (volumetric capacities of 918.3 and 829.4 mAh cm^−3^) after 240 cycles, with retentions of 75.3% and 73.4%, respectively (Figure 3h). Remarkably, even at an ultrahigh loading of 27.6 mg cm^−2^ (20 mA g^−1^) (Figure 3i), the electrode delivers a notable areal capacity of 11.4 mAh cm^−2^ and retains 58.8% of its capacity over 100 cycles (Figure 3j). Active material utilization remains high, reaching 79.3% at the maximum loading, benefiting from reduced polarization and enhanced kinetics at the lowered current density (Figure 3k). In summary, the 3D graphene encapsulation constructs an elastic conductive network that effectively mitigates alloy volume expansion, ensures fast charge transfer, and enables high electrode density. Consequently, HD‐Sb_0.6_Bi_0.4_@G simultaneously achieves high gravimetric/volumetric capacities, superior areal capacity, and excellent cycling stability—a comprehensive performance advantage surpassing most reported thick, dense electrodes (Figure 3l and Table S1).

To evaluate practical viability, a full cell (HD‐Sb_0.6_Bi_0.4_@G//PTCDA) was assembled (Figure 3m) [18, 46]. The perylene‐3,4,9,10‐tetracarboxylic dianhydride (PTCDA) cathode itself exhibits excellent stability, retaining 116.7 mAh g^−1^ after 100 cycles at 0.1 A g^−1^ (86% retention from the fifth cycle, Figure S9). The matched full cell demonstrates stable cycling at 0.2 A g^−1^, maintaining a high specific capacity of 277.6 mAh g^−1^ over 350 cycles with 60.6% capacity retention (Figure 3n). These results underscore the significant practical potential of the HD‐Sb_0.6_Bi_0.4_@G anode for high‐energy‐density PIBs [30].

### 2.3 Elucidating the Reaction Kinetics of Potassium‐Ion Storage in HD‐Sb_0.6_Bi_0.4_@G Monolithic Anodes

**FIGURE 4:**
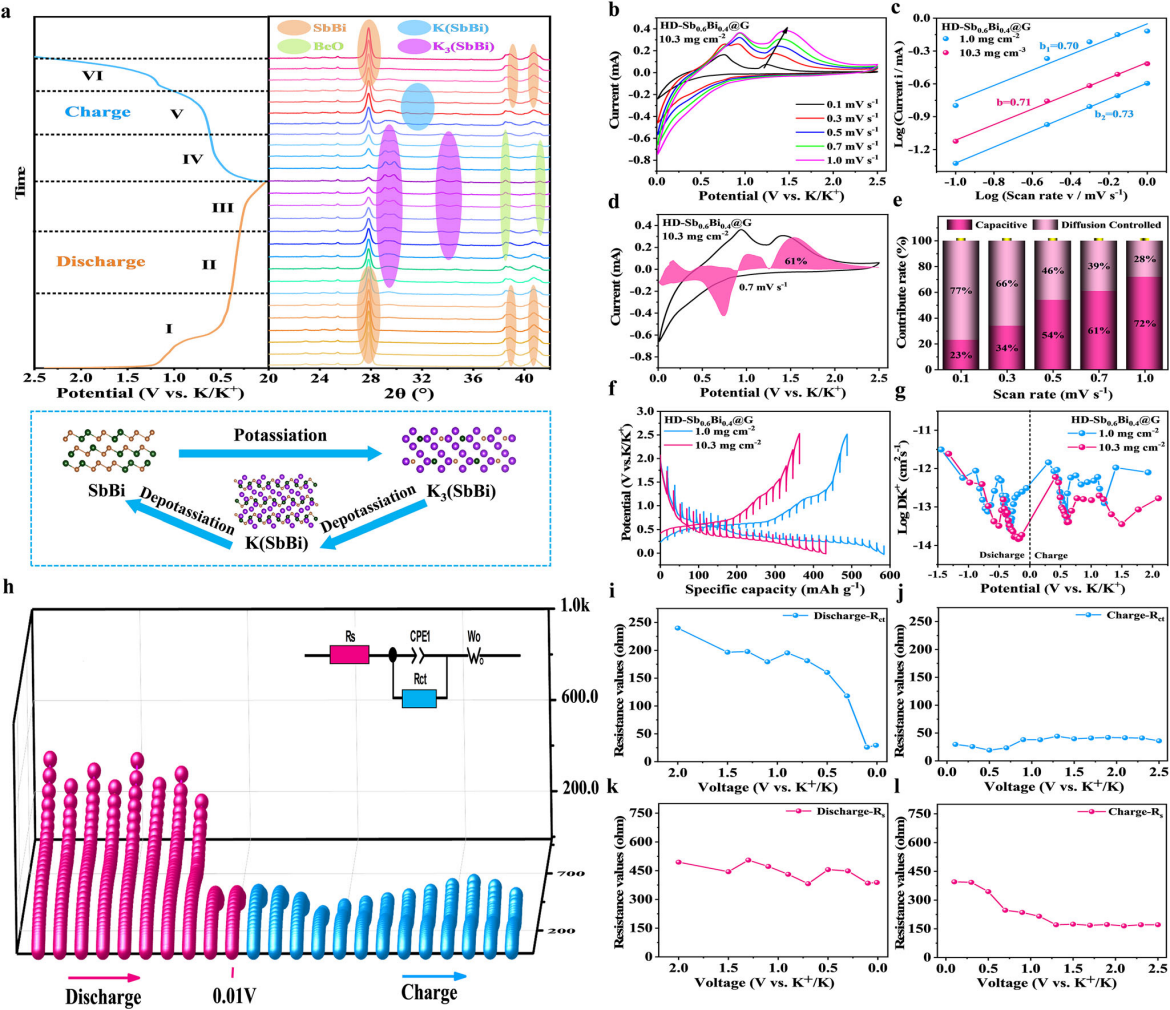
Mechanism and kinetics of potassium storage in a dense HD‐Sb_0.6_Bi_0.4_@G monolithic anode. (a) Initial discharge/charge curves of the HD‐Sb_0.6_Bi_0.4_@G anode with corresponding in situ XRD patterns at selected potentials, and a schematic of the potassiation/depotassiation process. (b) CV curves measured at various scan rates. (c) log(i) versus log(v) plots used for b‐value determination. (d) capacitive contribution at 0.7 mV s^−1^. (e) Normalized capacitive contribution ratios across different scan rates. (f) GITT potential profiles and (g) the corresponding calculated ionic diffusion coefficients. (h) Nyquist plots. (i,j) Charge transfer resistance (*R*_ct_) evolution during (I) discharge and (J) charge processes. (k,l) Solution resistance (R_s_) monitoring during (K) discharge and (L) charge cycles.

To investigate the K^+^ storage behavior of the HD‐Sb_0.6_Bi_0.4_@G anode, the initial potassiation process was tracked using in situ XRD [47]. The measurements were conducted at a current density of 0.1 A g^−1^. Figure 4a presents the resulting data, with the voltage‐time curve shown on the left and the corresponding XRD patterns at selected voltages displayed on the right. Upon discharge, the diffraction peaks of the SbBi alloy exhibit a gradual weakening between the open circuit voltage and 0.39 V, with no additional peaks emerging. The diffraction peaks of K_3_(SbBi) first appeared when the discharge was continued to 0.28 V, and subsequently, the peaks gradually strengthened during the discharge to 0.01 V. This indicates that the conversion of SbBi to K_3_(SbBi) was carried out in this process, and that the final state of the fully embedded potassium was K_3_(SbBi). Subsequently, during the charging process, the XRD spectra demonstrated that K_3_(SbBi) was the dominant phase before 0.62 V, while K_3_(SbBi) disappeared and diffraction peaks of K(SbBi) became apparent in the voltage interval of 0.62–1.05 V [48]. This indicates that K(SbBi) underwent depotassiumation to produce K(SbBi) in this voltage interval. During the subsequent charging process, only the diffraction peaks of SbBi were observed, and they gradually strengthened, indicating that K(SbBi) was further depotassiumated to the SbBi phase. From this, the initial potassium embedding and depotassiumation process of SbBi can be analyzed, and the transformation process of its phase structure can be summarized as SbBi→K_3_(SbBi)→K(SbBi)→SbBi [32].

To probe the potassium storage kinetics of HD‐Sb_0.6_Bi_0.4_@G electrodes under different mass loadings, CV was performed at various scan rates. The measurements were carried out on both low‐mass‐loading (1 mg cm^−2^) and high‐mass‐loading (10.3 mg cm^−2^) electrodes. The resulting CV profiles for the thin electrode (1 mg cm^−2^) are presented in Figure S10A, the results of thick electrodes are presented in Figure 4b. Upon increasing the scan rate from 0.1 to 1 mV s^−1^, a corresponding rise in peak current was observed for all electrodes. The absence of any substantial peak position shift in the thin electrode is indicative of a highly facile reaction with minimal electrochemical polarization. This indicates that ion and charge migration in HD‐Sb_0.6_Bi_0.4_@G is rapid. The shift of the peak position in thick electrodes is an unavoidable consequence of polarization and hysteresis, resulting from the increased electrode thickness [41, 45].

The relationship between the natural logarithms of the current (*i*) and the scanning rate (v) was obtained for the main peak current (*i*) and scanning rate (*v*) of the CV curves based on the equation *i = av^b^*, where a is a constant and b is the diffusion contribution (b = 0.5) and the pseudo‐capacitance contribution (b = 1) [24]. The results are shown in Figure 4c. The b‐values of the two peaks for the thin electrodes of HD‐Sb_0.6_Bi_0.4_@G were 0.70 and 0.73 for the thin electrode and 0.71 for the thick electrode. These values fall within the range of 0.5–1, indicating that both diffusion and pseudocapacitance contribute to the kinetic behaviors observed in both the thin and thick electrodes. The contribution of pseudocapacitance in HD‐Sb_0.6_Bi_0.4_@G was further investigated at different scan rates using the following equation *i(v) = k_1_v+k_2_v^1/2^*, where *k_1_v and k_2_v^1/2^* denote the pseudocapacitance contribution and diffusion control, respectively [49]. As illustrated in Figure S10B, the pseudocapacitance contributions of the HD‐Sb_0.6_Bi_0.4_@G thin electrodes at a scan rate of 0.7 mV s^−1^ are 70%. Figure 4d the pseudocapacitance contributions of the HD‐Sb_0.6_Bi_0.4_@G thick electrodes at a scan rate of 0.7 mV s^−1^ are 61%. This indicates that the two groups of electrodes are subjected to a greater contribution of pseudocapacitance behavior. Moreover, as illustrated in Figure S10C, the pseudocapacitance contribution of the HD‐Sb_0.6_Bi_0.4_@G thin electrode exhibits a notable increase from 53% to 82% with an elevated scan rate, whereas that of the thick electrode rises from 23% to 72% are shown in Figure 4e. It can be proposed that the HD‐Sb_0.6_Bi_0.4_@G thick electrode exhibits comparable rapid kinetics to the thin electrode. Furthermore, the pseudocapacitive contributions of the 2D Sb_0.6_Bi_0.4_ and 2D Sb_0.6_Bi_0.4_+G electrodes were evaluated. As illustrated in Figure S11A–G, the calculated b‐values for the two redox peaks of the 2D Sb_0.6_Bi_0.4_ electrode were 0.64 and 0.79, while those for the 2D Sb_0.6_Bi_0.4_+G composite were 0.76 and 0.80. All values fall within the range of 0.5–1, indicating a significant pseudocapacitive contribution to the total charge storage. Furthermore, their pseudocapacitive contributions at each scan rate are nearly identical to those of the high‐mass‐loading HD‐Sb_0.6_Bi_0.4_@G electrode (10 mg cm^−2^). These results demonstrate that even when 2D Sb_0.6_Bi_0.4_ is restructured into a 3D high‐density architecture, it retains the rapid kinetics inherent to its 2D counterpart. Moreover, the thick HD‐Sb_0.6_Bi_0.4_@G electrode maintains similar kinetic behavior and electrochemical performance to thin electrodes.

The K^+^ diffusion coefficients (*D*_K+_) in HD‐Sb_0.6_Bi_0.4_@G electrodes with varying loadings were subsequently determined by the galvanostatic intermittent titration technique (GITT) method. Each cycle involved a 10‐min charge/discharge pulse at 0.1 A g^−1^ and a subsequent relaxation step lasting 1 h [30, 42]. As illustrated in Figure 4f, the GITT curves of the two groups of electrodes exhibit comparable plateau characteristics to those observed in the constant‐current charge/discharge curves. The calculation results, detailed in Method S4, are presented in Figure 4g, the diffusion coefficients of K^+^ at each stage of the clearance results show that HD‐Sb_0.6_Bi_0.4_@G the thicker electrode exhibits a fast kinetic behavior similar to that of the thinner electrode. A comparison between the 2D Sb_0.6_Bi_0.4_ and 2D Sb_0.6_Bi_0.4_+G electrodes is presented in Figure S11H,I. Notably, the estimated diffusion coefficients reveal analogous kinetics not only in the 2D and thin‐film electrodes but also in the thick HD‐Sb_0.6_Bi_0.4_@G configuration. This provides direct evidence that the high‐mass‐loading electrode maintains exceptionally fast kinetics. This is mainly due to the high electrical conductivity HD‐Sb_0.6_Bi_0.4_@G can promote rapid electron transfer, the internal 2D Sb_0.6_Bi_0.4_ is completely enclosed by conductive graphene elastic network, maintaining a high specific surface area, can provide a large number of K^+^ contact sites, and 2D Sb and Bi nanolamellar structures may form new crystal structures. It provides more channels and space for K^+^ embedding, which is conducive to K^+^ transmission and rapid diffusion of K^+^.

Additionally, contact angle measurements in Figure S12 confirm the rapid wettability of the HD‐Sb_0.6_Bi_0.4_@G electrode toward the electrolyte. Upon contact with the HD‐Sb_0.6_Bi_0.4_@G electrode, the electrolyte rapidly wets the electrode surface, and the contact angle is almost 0°. This indicates that the HD‐Sb_0.6_Bi_0.4_@G electrode possesses excellent electrolyte wetting and penetration properties, which presents a promising opportunity for the HD‐Sb_0.6_Bi_0.4_@G electrode to be used as a substrate for the HD‐Sb_0.6_Bi_0.4_ electrode. The presence of Sb_0.6_Bi_0.4_@G in the electrode provides the foundation for the electrode to exhibit rapid ion diffusion behavior, which is a key attribute of HD‐Sb_0.6_Bi_0.4_@G.

The proposed electrochemical reaction mechanism was further confirmed through in situ EIS measurements [50]. As shown in Figure 4h–l, during the initial discharge process, the charge transfer resistance (*R*_ct_) of the HD‐Sb_0.6_Bi_0.4_@G electrode, as derived from EIS, exhibits a decreasing trend. This reduction can be attributed to the formation of highly conductive K_3_(SbBi) alloy via K^+^ intercalation. That is SbBi→K_3_(SbBi). In the process of insulation removal, the *R*_ct_ value follows the opposite trend, first decreasing and then increasing, which can be attributed to the K^+^ intercalation reaction resulting in K(SbBi) and the depotassium transformed into SbBi phase, namely K_3_(SbBi)→K(SbBi)→SbBi. The impedance variation rule and highly reversible electrochemical process are shown.

### 2.4 Unraveling the Potassium Storage Mechanism and Structural Stability of a Dense HD‐Sb_0.6_Bi_0.4_@G Monolithic Anode

**FIGURE 5:**
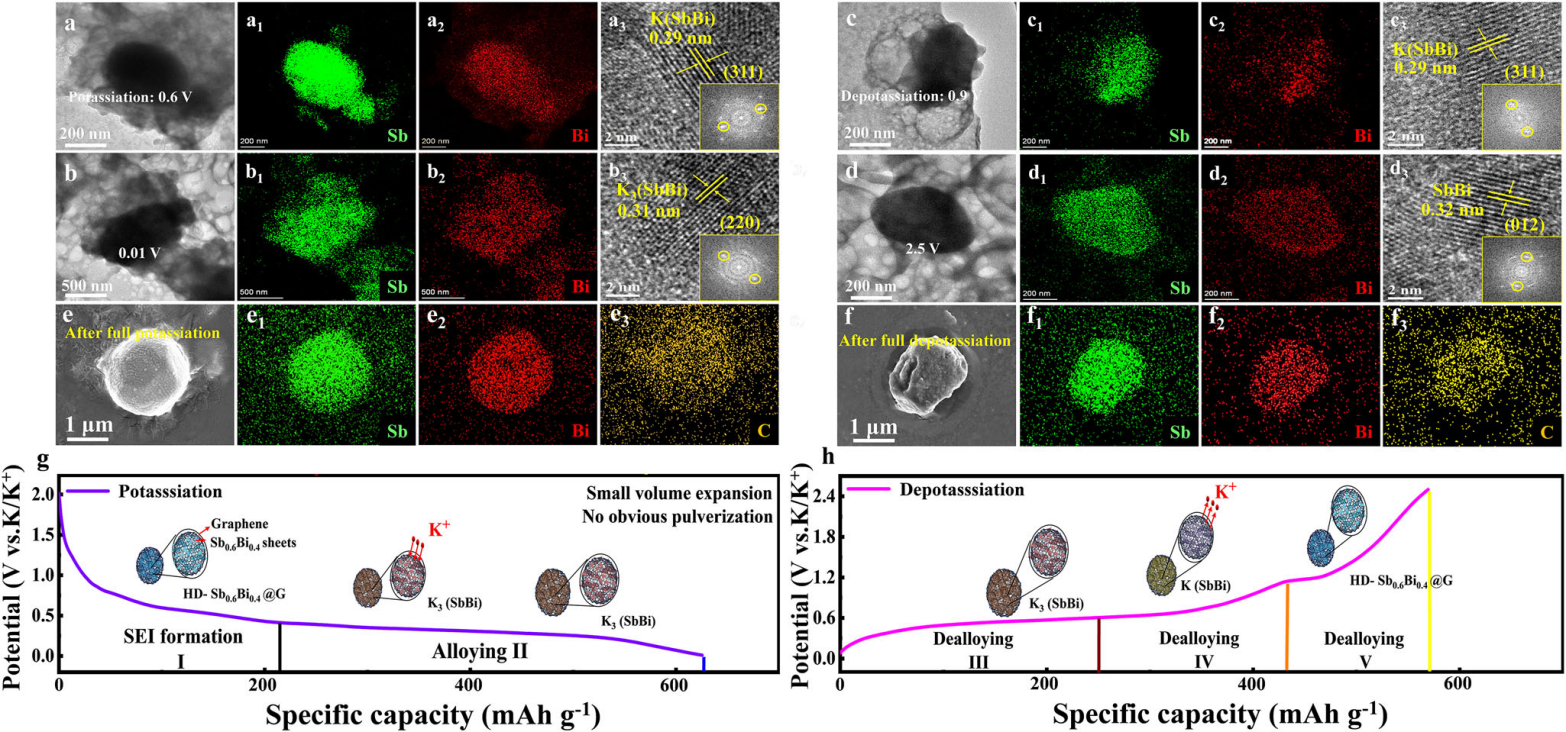
Ex situ TEM and SEM images of HD‐Sb_0.6_Bi_0.4_@G electrode. (a–d) Morphologies of the HD‐Sb_0.6_Bi_0.4_@G electrode at different (dis)charge states: (a) discharged to 0.6 V, (b) discharged to 0.01 V, (c) charged to 0.9 V, and (d) charged to 2.5 V. Corresponding elemental mapping (C, Sb, and Bi) and HRTEM images are included for each stage. (e,f) Ex situ SEM images after complete potassiation/depotassiation and the corresponding element distribution. (g,h) Structural evolution of the HD‐Sb_0.6_Bi_0.4_@G monolith during the cycling process.

To gain deeper insights into the morphological and structural changes of the HD‐Sb_0.6_Bi_0.4_@G anode during potassium ion intercalation and deintercalation, ex situ TEM analysis was conducted to examine its cycled morphology. The electrodes were charged or discharged to particular voltages after 200 cycles at 1 A g^−1^ and then characterized to capture their post‐cycling state. Following 200 cycles at 1 A g^−1^, ex situ TEM was performed on the HD‐Sb_0.6_Bi_0.4_@G electrode charged or discharged to specific voltages to examine its state. Figure 5a–d present the microstructural evolution of the HD‐Sb_0.6_Bi_0.4_@G anode at different potassium/depotassiation stages, as revealed by ex situ TEM. As observed in Figure 5a, when discharged to 0.6 V, the Sb_0.6_Bi_0.4_ alloy within the composite largely retains its original morphology. Furthermore, the Sb_0.6_Bi_0.4_@G nanosheets remain encapsulated within thin graphene layers, as clearly depicted in Figure 5a1,a2. HRTEM images clearly show the (311) crystal face of K(SbBi) at discharge to 0.6 V, indicating the presence of a transition from SbBi to K(SbBi) (Figure 5a3). Figure 5b reveals that the structural integrity of the Sb_0.6_Bi_0.4_ nanosheets remains largely intact even after full discharge to 0.01 V, corresponding to the complete potassiation stage (Figure 5b1,b2). The appearance of (110) crystal face streaks of K_3_(SbBi) in HRTEM images indicates that the final potassium product of Sb_0.6_Bi_0.4_ is K_3_(SbBi) (Figure 5b3) [32, 42].

Figure 5c displays the TEM image of HD‐Sb_0.6_Bi_0.4_@G charged to 0.9 V, in which the Sb_0.6_Bi_0.4_@G nanosheets retain their structural integrity (Figure 5c1,c2). The emergence of distinct lattice fringes corresponding to the (311) crystal plane of K(SbBi) in the HRTEM image (Figure 5c3) indicates the formation of K(SbBi) through the depotassiation of K_3_(SbBi). Meanwhile, Figure 5d corresponds to the fully charged state at 2.5 V, representing the complete depotassiation stage. The structural integrity of Sb_0.6_Bi_0.4_ nanosheets remained good (Figure 5d1,d_2_), indicating that Sb_0.6_Bi_0.4_ still had excellent crystallinity after depotassiumization. The presence of (012) lattice fringes from (SbBi) in the HRTEM image confirms that the depotassiation of K(SbBi) yields SbBi (Figure 5d3) [32, 42].

In the HD‐Sb_0.6_Bi_0.4_@G monolith, the elastic graphene network densely encapsulates the 2D Sb_0.6_Bi_0.4_ nanosheets, which effectively accommodates the mechanical strain from volume expansion during cycling. Therefore, the thickness change of the HD‐Sb_0.6_Bi_0.4_@G electrode before and after potassiation was investigated by ex situ SEM. At a mass loading of 1 mg cm^−2^, the 2D Sb_0.6_Bi_0.4_ electrode had a thickness of 10.3 µm (Figure S13A,a1). The electrode exhibited a thickness of 14.8 µm after potassiation, indicating a substantial volume swelling of 43.7%. In comparison, the original thickness of the HD‐Sb_0.6_Bi_0.4_@G electrode, at the same low loading level, exhibited a thickness of only 4 µm, which is indicative of a higher electrode density. Following the embedding of the full potassium, the electrode thickness was 4.9 µm, corresponding to a low volume expansion of 22.5% (Figure S13B,b1). This result confirms that the elastic encapsulation network effectively suppresses the volume expansion of the Sb_0.6_Bi_0.4_ nanoflakes, thus contributing to enhanced electrochemical stability. Further analysis was conducted to examine the volume changes in the HD‐Sb_0.6_Bi_0.4_@G thick electrode. At a high mass loading of 10.3 mg cm^−2^, the HD‐Sb_0.6_Bi_0.4_@G electrode exhibited a thickness increase from 39.5 to 47.2 µm after cycling, exhibiting a minimal volume expansion of only 19.5% (Figure S13C). As shown in Figure S13D, even at the highest mass loading of 27.6 mg cm^−2^, the electrode exhibited a thickness of 102.5 µm in the pristine state and 121.3 µm after complete potassiation, corresponding to a low volume expansion of only 18.3%. These results demonstrate that HD‐Sb_0.6_Bi_0.4_@G maintains excellent structural stability even in ultra‐thick electrode configurations, thereby enabling stable electrochemical performance during repeated cycling.

The robust electrode material contributes to maintaining a more stable monolithic structure. Therefore, the surface morphology of HD‐Sb_0.6_Bi_0.4_@G after potassiation/depotassiation was further examined via ex situ SEM, with results presented in Figure 5e,f. The images show electrode surface particles of HD‐Sb_0.6_Bi_0.4_@G in the fully discharged or charged state after rate cycling. It can be observed that the material maintains a dense microsphere structure without any cracking or pulverization, regardless of whether it is in the fully potassiated or depotassiated state.

The structural evolution during cycling further demonstrates the ability of the HD‐Sb_0.6_Bi_0.4_@G monolith to retain its structural integrity (Figure 5g,h). Throughout the potassiation/depotassiation process, the HD‐Sb_0.6_Bi_0.4_@G monolith undergoes slight volume expansion while maintaining structural coherence. Due to the graphene encapsulation, the dense HD‐Sb_0.6_Bi_0.4_@G remains intact without fracture even after complete potassium insertion/extraction. This stable architecture establishes the foundation for achieving ultra‐stable electrochemical cycling in PIBs. These results reaffirm that the elastic graphene network effectively buffers volume variations of Sb_0.6_Bi_0.4_ during cycling, endowing the HD‐Sb_0.6_Bi_0.4_@G monolith with exceptional structural robustness and thereby preserving high structural integrity throughout repeated cycling processes.

### 2.5 Comparative DFT Study on HD‐Sb@G, HD‐Bi@G, and HD‐Sb_0.6_Bi_0.4_@G Anodes for Potassium‐Ion Storage Mechanism

**FIGURE 6:**
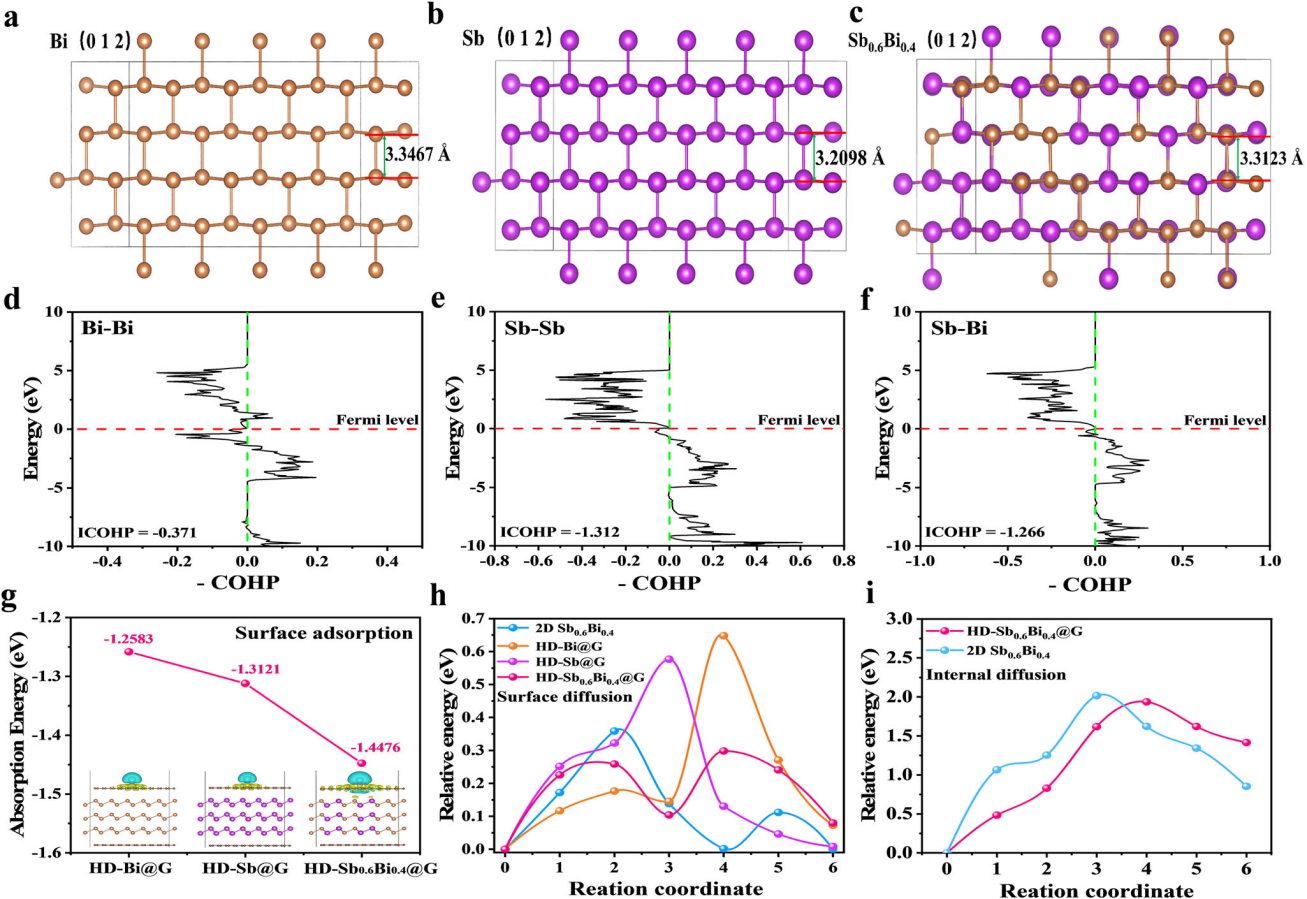
DFT calculation of HD‐Sb_0.6_Bi_0.4_@G anodes in PIBs. (a) Interlayer spacing of the (012) crystal plane in Bi. (b) Sb, and (c) Sb_0.6_Bi_0.4_. (d) Bi─Bi, (e) Sb─Sb, and (f) Sb─Bi bond energy. (g) Comparison of surface adsorption energy barriers among HD‐Sb@G, HD‐Bi@G, and HD‐Sb_0.6_Bi_0.4_@G. (h) Comparison of surface diffusion energy barriers among 2D Sb_0.6_Bi_0.4_, HD‐Sb@G, HD‐Bi@G, and HD‐Sb_0.6_Bi_0.4_@G. (i) Comparison of the inner diffusion energy barriers of 2D Sb_0.6_Bi_0.4_ and HD‐Sb_0.6_Bi_0.4_@G.

We performed DFT calculations to investigate the mechanism behind the exceptional potassium storage performance of the HD‐Sb_0.6_Bi_0.4_@G anode for PIBs. The substantial ionic radius of K^+^ (∼1.38 Å) necessitates a moderately expanded interlayer spacing to reduce diffusion energy barriers and mitigate volumetric strain during cycling. As shown in Figure 6a–c, the interlayer spacings of the (012) crystal planes were compared: 3.2098 Å for 2D Sb, 3.3467 Å for 2D Bi, and an intermediate value of 3.3123 Å for 2D Sb_0.6_Bi_0.4_, which is closer to that of Sb. Notably, while an excessively large interlayer spacing benefits ion migration, it may compromise structural stability or electrical conductivity [32, 33]. To investigate this balance mechanism, the bond energies of Sb─Sb, Bi─Bi, and Sb─Bi were calculated and compared (Figure 6d–f) [36, 37]. The results demonstrate that the Sb─Sb bond possesses the highest strength (ICOHP = −1.312 eV), the Bi─Bi bond the weakest (ICOHP = −0.371 eV), and the Sb─Bi bond exhibits an intermediate strength (ICOHP = −1.266 eV). Consequently, the 2D Sb_0.6_Bi_0.4_ alloy achieves an optimal balance between interlayer spacing and bond energy: its relatively large interlayer spacing ensures rapid K^+^ diffusion kinetics, while the moderate bond energy enables facile K^+^ deintercalation while maintaining structural stability. This unique alloying effect is key to optimizing the balance between diffusion kinetics and structural stability.

Second, the areal density of active sites on 2D alloy electrodes governs their electrochemical performance. As illustrated in Figure 6g, DFT calculations were employed to determine the K^+^ adsorption capacity at active sites for HD‐Sb@G, HD‐Bi@G, and HD‐Sb_0.6_Bi_0.4_@G after structural optimization. HD‐Sb@G exhibits a higher K^+^ adsorption energy (−1.3121 eV) at surface atomic sites (Site 1) compared to HD‐Bi@G (−1.2583 eV), while HD‐Sb_0.6_Bi_0.4_@G demonstrates the highest adsorption energy (−1.4476 eV). By integrating a 3D graphene encapsulation model and analyzing charge density differences among HD‐Sb@G, HD‐Bi@G and HD‐Sb_0.6_Bi_0.4_@G, it is evident that the synergistic interaction between Sb and Bi, coupled with the compact graphene network, significantly enhances the overall adsorption energy in HD‐Sb_0.6_Bi_0.4_@G. This unique architecture leads to superior potassium adsorption and storage capabilities.

Furthermore, the K^+^ diffusion rate within the electrode material governs the rate capability of PIBs [32, 33, 42]. DFT computations were conducted on these systems. The aim was to probe the energy barriers associated with K^+^ surface diffusion. Figure 6h compares the K^+^ surface diffusion barriers across the studied materials: HD‐Sb@G (0.5767 eV) exhibits a lower barrier than HD‐Bi@G (0.6479 eV), while 2D Sb_0.6_Bi_0.4_ (0.3586 eV) shows an even lower barrier. Remarkably, HD‐Sb_0.6_Bi_0.4_@G achieves the lowest diffusion barrier (0.2982 eV), surpassing all other materials. These results indicate that the optimized interlayer spacing and bond energy of the Sb_0.6_Bi_0.4_ alloy, combined with the synergistic effects of alloying and the 3D graphene encapsulation, provide additional channels and space for K^+^ migration, facilitating easier intercalation/deintercalation. Since the electrochemical process involves both surface and subsurface K^+^ diffusion, the diffusion barriers in the second atomic layer of 2D Sb_0.6_Bi_0.4_ and HD‐Sb_0.6_Bi_0.4_@G were also calculated. As depicted in Figure 6i, HD‐Sb_0.6_Bi_0.4_@G (1.6224 eV) maintains a lower barrier than 2D Sb_0.6_Bi_0.4_ (1.9365 eV), consistent with the surface trend.

High‐rate charging/discharging requires both rapid ion diffusion and efficient electron transport. Thus, the band structures and density of states (DOS) of HD‐Sb@G, HD‐Bi@G, and HD‐Sb_0.6_Bi_0.4_@G were calculated and compared. As shown in Figure S14A–C, the band structures of HD‐Sb@G, HD‐Bi@G and HD‐Sb_0.6_Bi_0.4_@G reveal no bandgap, confirming retained metallic conductivity. As shown in Figure S14D–F, the analysis of the DOS distribution demonstrates that HD‐Sb_0.6_Bi_0.4_@G exhibits modified electronic states near the Fermi level compared to HD‐Sb@G and HD‐Bi@G [51, 52]. The alloy shows nearly equal contributions from Sb and Bi to the conduction and valence bands, resulting in enhanced electronic conductivity. This improvement, coupled with the optimized ionic diffusion pathways, synergistically boosts the rate capability.

## 3 Conclusion

In summary, we developed a synergistically intrinsic‐extrinsic dual‐stabilization strategy to engineer a dense monolithic architecture (HD‐Sb_0.6_Bi_0.4_@G) by integrating 2D alloy nanosheets into the 3D elastic graphene network. This architecture yields a high density (2.6 g cm^−3^), excellent conductivity (555.6 S m^−1^), and robust mechanical buffering. The HD‐Sb_0.6_Bi_0.4_@G anode exhibits superior volumetric (1355.1 mAh cm^−3^) and areal (11.4 mAh cm^−2^) capacities, with outstanding long‐term cycling stability (65.4% retention after 1500 cycles at 1 A g^−1^). Such high volumetric and areal capacities outperform previously reported most PIB anodes. When paired with a PTCDA cathode, the full cell maintains 60.6% capacity over 350 cycles at 0.2 A g^−1^. Such excellent results mainly stem from the following three aspects: (1) The Sb─Bi alloy exhibits “bond softening” with optimized interlayer spacing and moderated bond energy, which facilitates rapid K^+^ diffusion and intrinsic stability. (2) The elastic 3D graphene network extrinsically accommodates volume expansion and prevents electrode degradation. (3) The strong electronic coupling at the alloy‐graphene interface further reduces K^+^ adsorption and diffusion barriers, enabling fast ion transport under high‐loading electrodes. Therefore, this intrinsic‐extrinsic synergy between bond softening of binary alloys and nanoscale elastic confinement provides a universal strategy for compact, high‐loading electrodes with high volumetric and areal performance.

## Conflicts of Interest

The authors declare no conflicts of interest.

## Supporting information




**Supporting File 1**: anie71970‐sup‐0001‐SuppMat.docx.

## Data Availability

The data that support the findings of this study are available from the corresponding author upon reasonable request.
