# Supplementary material for: Bond Softening and Elastic Confinement of Monolithic Dense Binary Alloys for High Volumetric Capacity of K‐Ion Battery Anodes
Source: Angew Chem Int Ed Engl. 2026 Mar 23;65(19):e9686588. doi: 10.1002/anie.9686588 (PMC13134609; doi:10.1002/anie.9686588)
Supplement: Supplementary file 1 — Supporting File 1: anie71970‐sup‐0001‐SuppMat.docx. [file ANIE-65-e9686588-s001.docx]

**Supporting Information**

**Bond Softening and Elastic Confinement of Monolithic Dense** **Binary Alloys for High Volumetric Capacity of K-Ion Battery Anodes**

*Yunyong Li^1 ,^**^3^, Yiru Zhou^3^,* *Bingchun Wang^3^,* *Xinying Wang^3^,* *Lei Liu^3^,* *Zhuhang Shao^3^,* *Hao Wu^3^,* *Zaowen Zhao^1^*, *Yingqiang Wu^1^,* *Xuerong Zheng^1,^*,* *Xinwei Li*^2^*^,^**, *Wenwu Li*^2^, *Yida Deng**^1,^*,* *Zaiping Guo^4^, and Ho Seok Park^2, 5,^**

^1^State Key Laboratory of Tropic Ocean Engineering Materials and Materials Evaluation, School of Materials Science and Engineering, Hainan University, Haikou, 570228, PR China

^2^School of Chemical Engineering, Sungkyunkwan University, 2066, Seoburo, Jangan-gu, Suwon, 440-746, South Korea

^3^School of Materials and Energy, Guangdong University of Technology, No. 100 Waihuan Xi Road, Guangzhou Higher Education Mega Centre, Guangzhou, 510006, PR China

^4^School of Chemical Engineering, The University of Adelaide, Adelaide 5000, Australia

^5^SKKU Institute of Energy Science and Technology (SIEST), Sungkyunkwan University (SKKU), 2066, Seobu-ro, Jangan-gu, Suwon, Gyeonggi-do 16419, Republic of Korea

*Correspondence: xrzh@hainanu.edu.cn (X. R. Zheng); [lixinwei2580@skku.edu](mailto:lixinwei2580@skku.edu) (X. W. Li); [yd_deng@hainanu.edu.cn](mailto:yd_deng@hainanu.edu.cn) (Y. D. Deng); [phs0727@skku.edu](mailto:phs0727@skku.edu) (H. S. Park)

**Section Ⅰ. Experimental Sections**

**Synthesis of 2D Sb_0.6_Bi_0.4_ Alloy**. A solution was prepared by dissolving antimony trichloride (SbCl_3_, 6 mmol) and bismuth trichloride (BiCl_3_, 4 mmol) in 200 mL of ethanol. Then, 100 mL of 2 M hydrochloric acid (HCl) was added under continuous stirring. Subsequently, iron powder (11 mmol) was gradually introduced and dispersed into the mixture, which was left to react for 48 hours. Upon completion, residual iron was removed via treatment with hydrochloric acid. The final product was collected via vacuum filtration, meticulously rinsed with ethanol and deionized water, and subsequently subjected to freeze-drying, yielding a two-dimensional Sb_0.6_Bi_0.4_ alloy powder.

(Fe + 0.6 Sb^3+^ + 0.4 Bi^3+^ → Sb_0.6_Bi_0.4_ + Fe^3+^)

**Synthesis of 2D Sb and 2D Bi**. The two-dimensional antimony (2D Sb) was synthesized employing a method analogous to that used for the Sb_0.6_Bi_0.4_ alloy, utilizing antimony trichloride (SbCl_3_, 10 mmol) as the exclusive metal precursor in the absence of BiCl_3_. The two-dimensional bismuth (2D Bi) was fabricated by first dissolving bismuth trichloride (BiCl_3_, 1.0 g) in 100 mL of N-methyl-2-pyrrolidone (NMP). Subsequently, 5 mL of 4 M HCl was added dropwise under constant stirring. Following this, iron powder (0.2 g) was introduced gradually, and the mixture was allowed to react undisturbed for 48 hours. The resulting product was isolated via filtration, thoroughly washed with ethanol and deionized water, and finally freeze-dried to obtain the desired 2D Bi material.

(2Sb^3+^ + 3Fe → 2Sb + 3Fe^2+^), (2Bi^3+^ + 3Fe → 2Bi + 3Fe^2+^)

**Preparation of HD-Sb_0.6_Bi_0.4_@G**. A homogeneous dispersion was prepared by mixing 2D Sb_0.6_Bi_0.4_ powder (85 wt%) and graphene oxide (GO, 15 wt%) in N,N-dimethylformamide (DMF) at a concentration of 3 mg mL^-1^ using magnetic stirring and ultrasonication. The mixture was then transferred to a Teflon-lined autoclave and underwent solvothermal treatment at 200 °C for 24 h, resulting in the formation of an Sb_0.6_Bi_0.4_@reduced graphene oxide (RGO) hydrogel. The obtained hydrogel was dried at room temperature for 48 h to form a dense monolithic structure, which was subsequently annealed at 500 °C for 2 h under a H_2_/Ar atmosphere to yield the final product, denoted as HD-Sb_0.6_Bi_0.4_@G.

**For comparison, a physically blended composite, labeled 2D** Sb_0.6_Bi_0.4_**+G, was fabricated by thoroughly mixing 2D** Sb_0.6_Bi_0.4_ **alloy powder (85 wt%) with thermally reduced graphene (15 wt%).**

**Preparation of HD-Sb@G and HD-Bi@G**. HD-Sb@G was synthesized following the same procedure as HD-Sb_0.6_Bi_0.4_@G but using 2D Sb powder as the precursor. For HD-Bi@G, a dispersion containing 2D Bi (85 wt%) and GO (15 wt%) in DMF at a concentration of 4 mg mL⁻¹ was subjected to solvothermal treatment at 200 °C for 24 h, resulting in the formation of a Bi@RGO hydrogel. The hydrogel was dried to obtain a monolithic structure, which was subsequently annealed at 250 °C for 4 h under a H_2_/Ar atmosphere to yield the final HD-Bi@G composite.

**Section II** **Materials Characterizations**

The morphologies and microstructures of the samples were analyzed using field emission scanning electron microscopy (SEM, Hitachi SU8010) and transmission electron microscopy (TEM, FEI Tecnai G20). The crystal structures were characterized by powder X-ray diffraction (XRD) on a Rigaku D/Max-III diffractometer with Cu Kα radiation. Diffraction patterns were recorded over a 2*θ* range of 5° to 80° at a scan rate of 10° min^-1^. Raman scattering measurements were conducted at room temperature using a Horiba Jobin Yvon confocal Raman microscope equipped with a 532 nm laser as the excitation source. The elemental composition and chemical states of the samples were analyzed by X-ray photoelectron spectroscopy (XPS) using two separate systems with monochromatic Al Kα sources (ESCALab250, USA).

*In-situ* XRD characterization was conducted to monitor structural evolution using an Empyrean X-ray diffractometer (PANalytical B. V., the Netherlands) over a scanning angle range of 20°-42° at a scan rate of 2° min^-1^.

*Ex-situ* TEM was performed to investigate the morphological and structural evolution of the HD-Sb_0.6_Bi_0.4_@G electrodes at different potassiation/depotassiation stages. The electrodes were disassembled in an argon-filled glovebox (H_2_O and O_2_ < 0.1 ppm), and the active materials were scraped off, dispersed in anhydrous ethanol, and dropped onto holey carbon-coated copper grids. To minimize air exposure, the samples were transferred to the microscope using an airtight vacuum holder.

**Section ⅠII. ElectrodeFabrication and Assembly Details**

**Fabrication and Assembly of Electrodes for Coin-Type Half-Cells.** The working electrodes were fabricated by mixing the as-prepared composite materials (90 wt%) with a binder (10 wt%) without any additional conductive additives. An appropriate solvent was added, and the mixture was stirred to form a homogeneous slurry. This slurry was then coated onto a current collector and dried at 60 °C for 24 hours to remove the solvent. For the thin electrodes (mass loading: < 4 mg cm^-2^): the binder was a mixture of polyacrylic acid (PAA, purchased from Aladdin Co., Ltd.) and KOH, deionized water was used as the solvent, and conventional copper foil served as the current collector. For the thick electrodes (mass loading: > 4 mg cm^-2^): the slurry consisted of a binder mixture (PAA and KOH, 10 wt%) and HD-Sb_0.6_Bi_0.4_@G (90 wt%) with the corresponding solvent, and conventional copper foil was used as the current collector. Subsequently, the electrodes were assembled into coin cells (2032-type) using a glass fiber separator (Whatman, GF/D) and potassium metal as the counter electrode. The optimized electrolyte was 4.0 M potassium bis(fluorosulfonyl)imide (KFSI) in a mixture of ethylene carbonate (EC) and diethyl carbonate (DEC) (1:1 by volume). This electrolyte was selected for its ability to form a robust and stable solid electrolyte interphase (SEI), thereby contributing to superior electrochemical performance.

For comparison, the HD-Sb@G, HD-Bi@G, and 2D Sb_0.6_Bi_0.4_+G composite electrodes were prepared and assembled into cells following the identical procedures used for the HD-Sb_0.6_Bi_0.4_@G electrode. To facilitate a more comprehensive performance evaluation, an additional control electrode (denoted as the 2D Sb_0.6_Bi_0.4_ electrode) was prepared by thoroughly mixing 2D Sb_0.6_Bi_0.4_, conductive carbon black, and the binder (PAA and KOH mixture) in a weight ratio of 75:15:10 to form a homogeneous slurry. This slurry was also coated onto copper foil and dried at 60 °C for 24 hours. The cell assembly process for this control electrode was consistent with that of the HD-Sb_0.6_Bi_0.4_@G electrode.

**Fabrication and Assembly of Electrodes for Coin-Type Full Cells.** For the assembly of full cells, the cathode material employed was perylene-3,4,9,10-tetracarboxylic dianhydride (PTCDA), purchased from Aladdin. The preparation process was as follows: PTCDA was annealed at 450 °C for 4 hours under an argon atmosphere at a heating rate of 5 °C min^-1^. Subsequently, it was mixed with carbon black and sodium carboxymethyl cellulose (CMC) in a weight ratio of 7:2:1, and the resulting mixture was coated onto aluminum foil. In this potassium-ion full cell, the capacity ratio of the anode material (HD-Sb_0.6_Bi_0.4_@G) to the cathode material (PTCDA) was approximately 1:1.1-1.2. PTCDA and HD-Sb_0.6_Bi_0.4_@G served as the cathode and anode, respectively, in separate half-cells, where they underwent a pre-potassiation process consisting of 5 cycles at 0.1 A g^-1^.

**Section ⅠV. Calculation Methods**

**S1** **The calculation method for the volumetric density of HD-Sb_0.6_Bi_0.4_@G monolith and the compacted density of electrode.**

The volume density of HD-Sb_0.6_Bi_0.4_@G monolith was tested by the formula of *ρ=m/v*, where *m* and *v* is the weight and volume of HD-Sb_0.6_Bi_0.4_@G monolith, respectively. The monolith was cut into a cylinder, the weight is 0.238 g, length is 0.9 cm, and diameter is 0.4 cm, so the density of HD-Sb_0.6_Bi_0.4_@G monolith was calculated can up to 2.1 g cm^-3^.

The electrode with a loading of 10.3 mg cm^-1^ was used to calculate the compacted density to reduce the measurement error. Figure 6C shows the electrode (diameter: 10 mm, thickness: 39.5 μm, mass loading: 10.3 mg cm^-2^ (excluding the current collector)). After calculation, the compacted density of HD-Sb_0.6_Bi_0.4_@G electrode is around 2.6 g cm^-3^.

**S2 The conductivity calculation method of HD-Sb_0.6_Bi_0.4_@G monolith.**

The conductivity of HD-Sb_0.6_Bi_0.4_@G monolith was tested at the electrochemical workstation by *I*-*V* curves *via* the formula of *ρ=R×A/L*. (*ρ*: resistivity; *R*: resistance; *A*: bottom area; *L*: the length of HD-Sb_0.6_Bi_0.4_@G monolith).The HD-Sb_0.6_Bi_0.4_@G monolith was cut into a cylinder with the diameter of 0.4 cm, length of 0.9 cm, the voltage was set from -0.4-0.4 V. By calculation of the formula of *σ=1/ρ* (where electric conductivity is the reciprocal of resistivity), the conductivity of HD-Bi@G monolith is 555.6 S m^-1^.

**S3 Volumetric capacity calculation method for the dense HD-Sb_0.6_Bi_0.4_@G electrode.**

The volumetric capacity of the HD-Sb_0.6_Bi_0.4_@G electrode was calculated based on the whole volume of the compact electrode by the formula of *C_v_* (mAh cm^-3^) = *C_g_* (mAh g^-1^) × *ρ* (g cm^-3^). Among them, *Cv*, *Cg*, and *ρ* represent volumetric capacity, specific capacity of the whole electrode materials including binder, and the compacted density of the electrode, respectively. Herein, the HD-Sb_0.6_Bi_0.4_@G electrode at 1.0 A g^-1^ was used as an example (loading: 1.0 mg cm^-2^). The initial gravimetric capacity (*C_g_*) is 521.2 mAh g^-1^, while the compacted density (*ρ*) of the electrode is 2.6 g cm^-3^, thus the volumetric capacity (*C_v_*) calculated is *C_v_* (mAh cm^-3^) = *C_g_* (521.2 mAh g^-1^) × *ρ* (2.6 g cm^-3^) = 1355.1 mAh cm^-3^.

**S4 Calculation of potassium-ion diffusion coefficient based on GITT.**

The diffusion coefficient could be calculated by the formula $\text{D}_{\text{K}^{\text{+}}}\text{=}\frac{\text{4}}{\text{πτ}}{\text{(}\frac{\text{n}\text{V}_{\text{m}}}{\text{S}}\text{)}}^{\text{2}}{\text{(}\frac{{\text{∆}\text{E}}_{\text{s}}}{{\text{∆}\text{E}}_{\text{t}}}\text{)}}^{\text{2}}$, where *n* and *V_m_* represent the molar number and molar volume of the active material, *τ*, *S*, *E_s_*, and *E_t_* represent the relaxation time, macroscopic area of electrode/electrolyte contact, voltage change caused by the pulse, and voltage change caused by continuous current charge and discharge, respectively.

**S5 Density Functional Theory (DFT) Calculation.**

The present first-principle DFT calculations are performed by Vienna Ab initio Simulation Package (VASP) with the projector augmented wave (PAW) method. The exchange-functional is treated using the generalized gradient approximation (GGA) of Perdew-Burke-Ernzerhof (PBE) functional. After the test, the energy cutoff for the plane wave basis expansion is selected to be 450 eV, the Brillouin zone integration was sampled by 2*4*1 point and the force on each atom less than 0.05 eV/Å was set for convergence criterion of geometry relaxation. 15 Å vacuum was added along the z direction in order to avoid the interaction between periodic structures. The self-consistent calculations apply a convergence energy threshold of 10-5 eV. The DFT-D3 method was employed to consider the van der Waals interaction.

The adsorption energy of K^+^ was calculated according to

$$\text{E}_{\text{ads}}\text{=}\text{E}_{\text{total}}\text{-}\text{E}_{\text{sur}}\text{-}\text{E}_{\text{k}\text{+}}$$

Where *E*_total_ is the total energy of the K^+^ adsorbed systems, *E*_sur_ and *E*_K+_ are the energies of the surface (HD-Sb@G, HD-Bi@G, and HD-Sb_0.6_Bi_0.4_@G (001) surface) and the isolated K ion, respectively.

**Section V. Supplementary Figures and Tables**

**
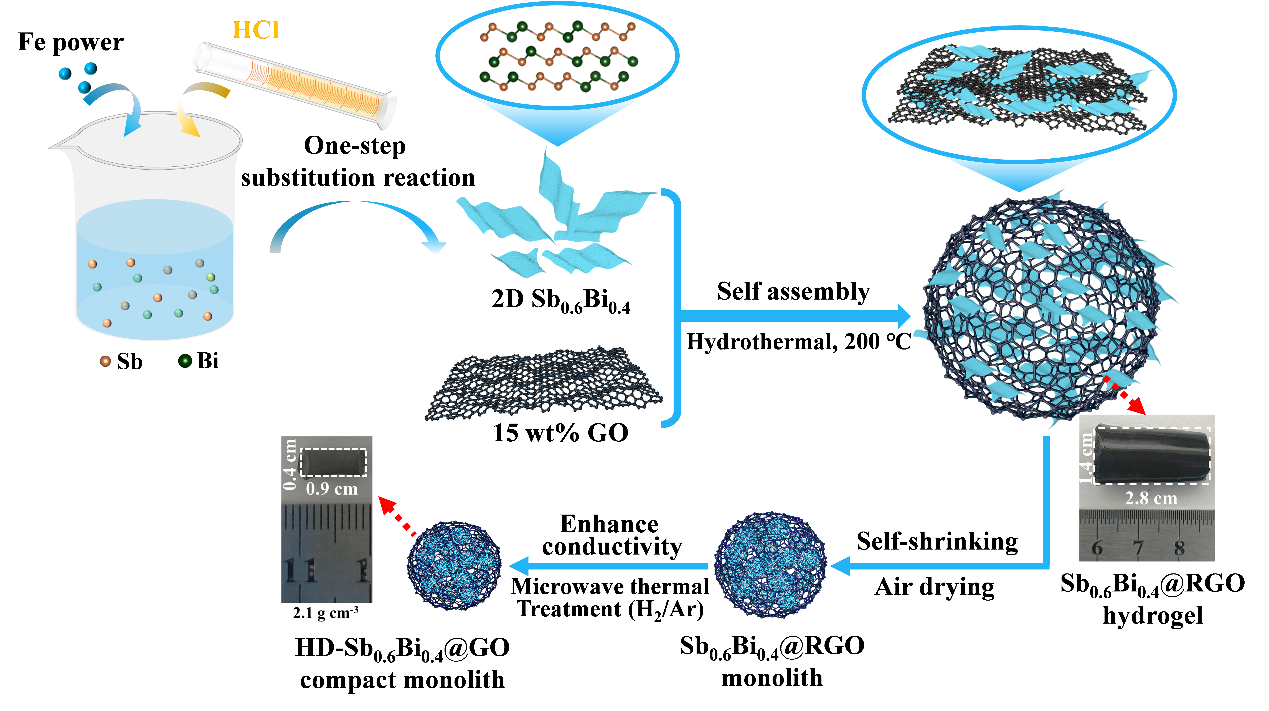
**

**Figure S1**. Schematic illustration of the synthesis processes for the HD-Sb_0.6_Bi_0.4_@G compact monolith.


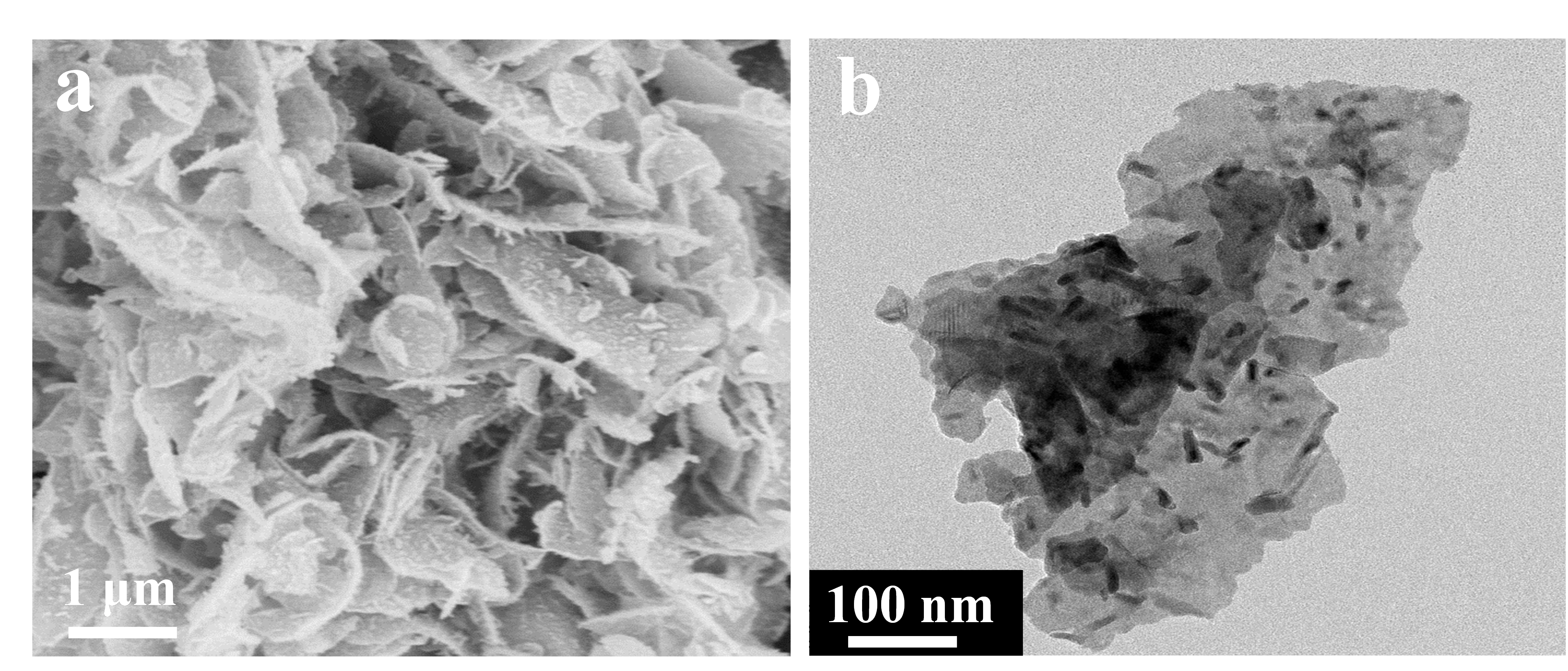


**Figure S2**. (a) SEM and (b) HAADF-STEM image of 2D Sb_0.6_Bi_0.4_.


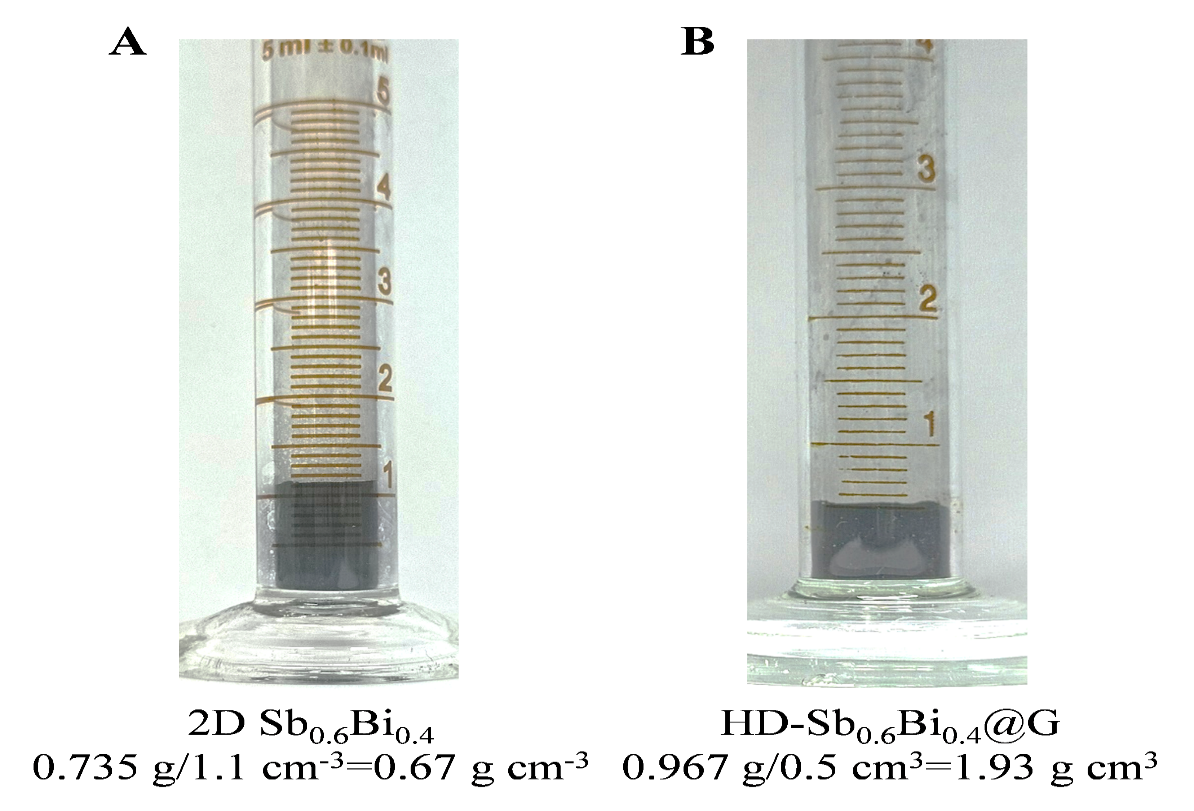


**Figure S3**. Digital photos of (A) 2D Sb_0.6_Bi_0.4_ and (B) HD-Sb_0.6_Bi_0.4_@G after tapping for 1000 times.


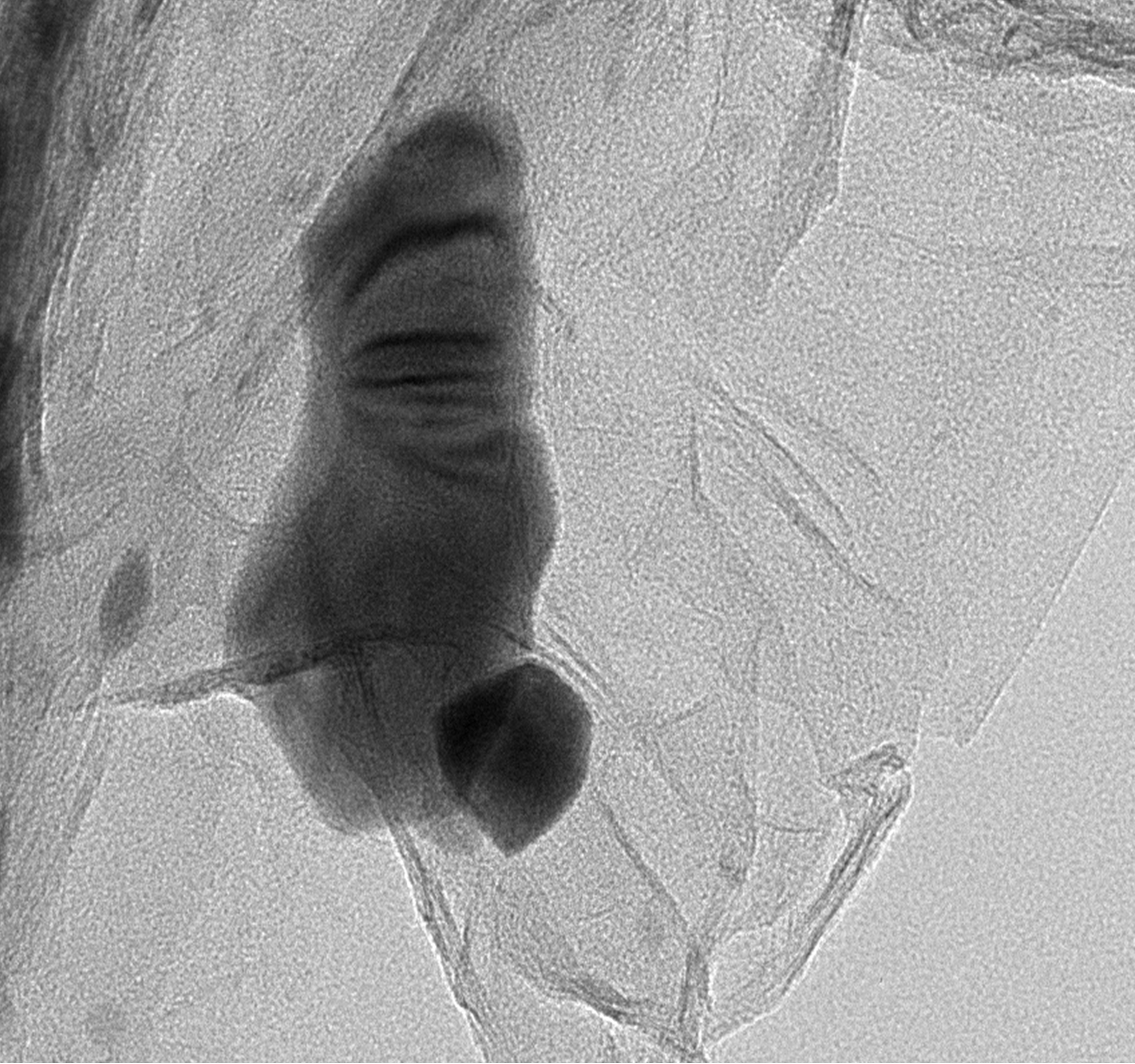


**Figure S4**. TEM image of HD-Sb_0.6_Bi_0.4_@G particles.


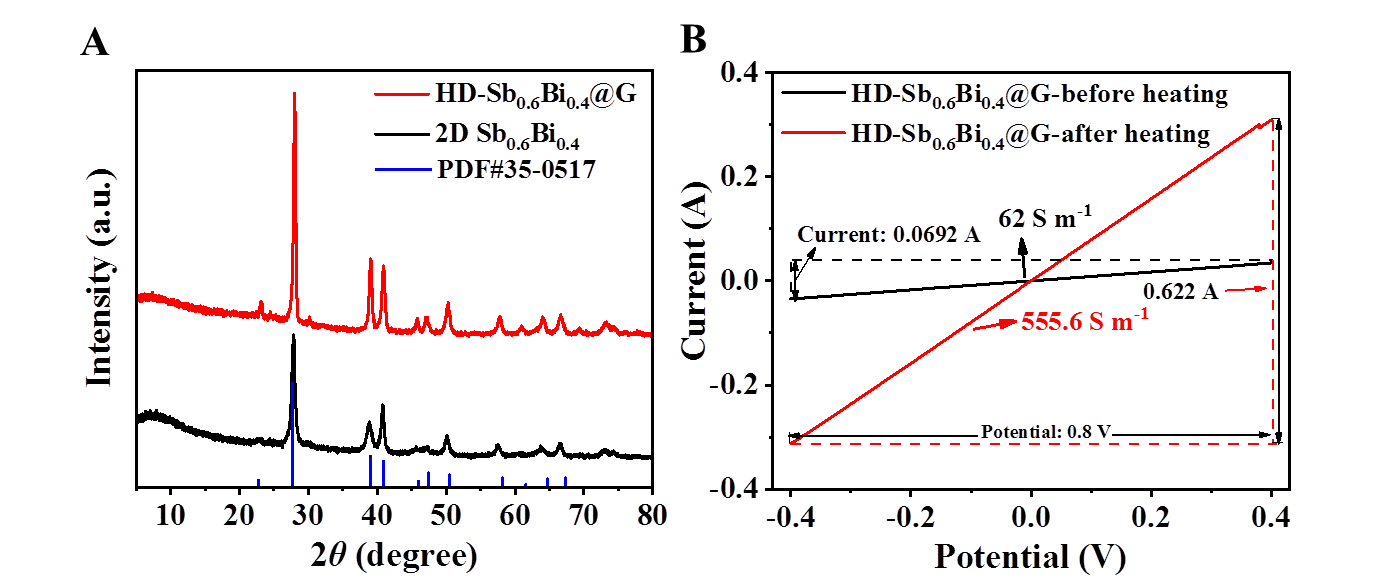


**Figure S5.** (A) **XRD curves of HD-Sb_0.6_Bi_0.4_@G and 2D Sb_0.6_Bi_0.4_.** (B) Conductivity test of HD-Sb_0.6_Bi_0.4_@G monolith (before and after heating).


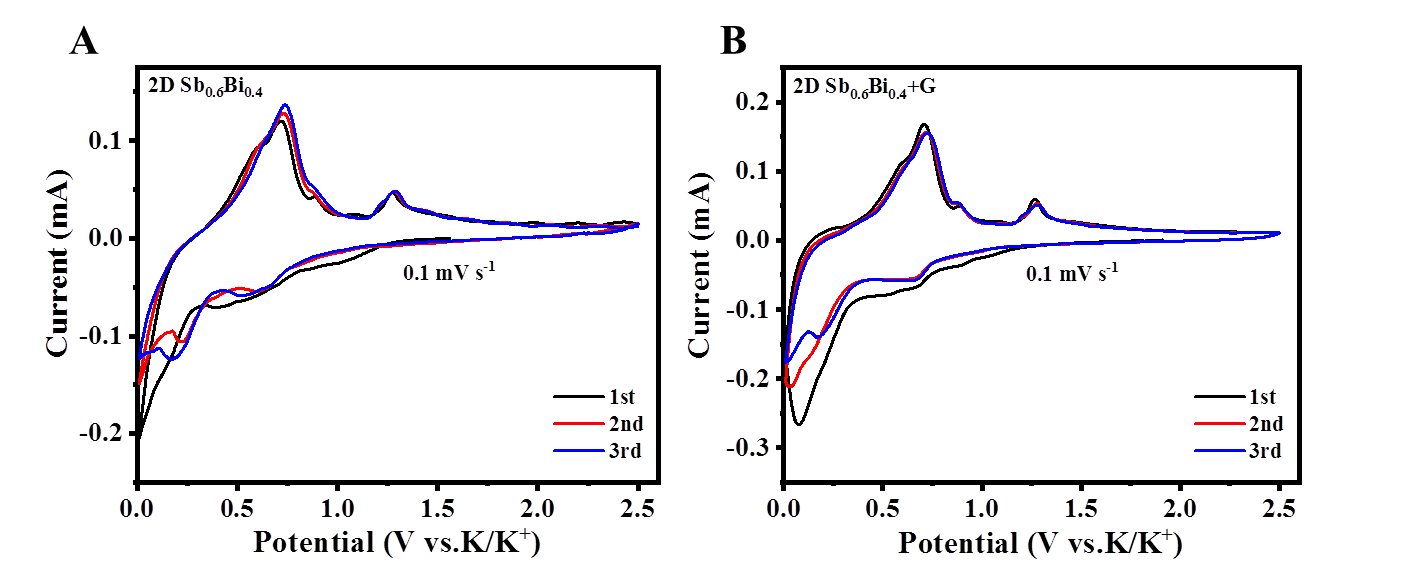


**Figure S6**. CV curves of 2D **Sb_0.6_Bi_0.4_**, 2D **Sb_0.6_Bi_0.4_**+G anodes and their comparison.


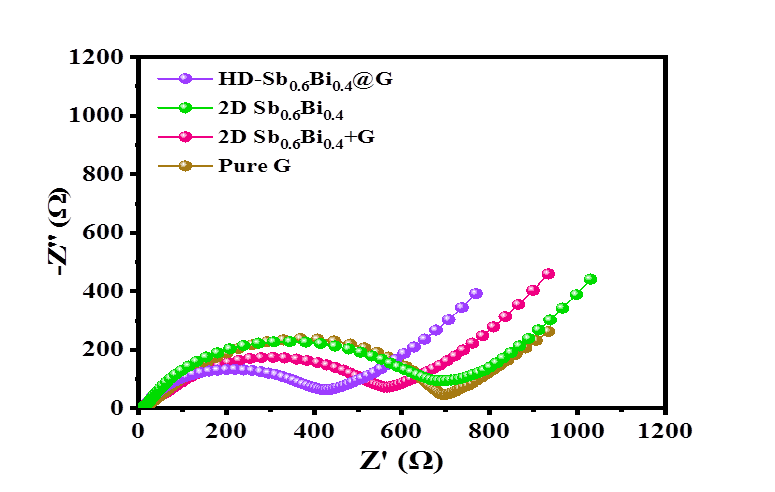


**Figure S7**. Nyquist plots of pure G, 2D **Sb_0.6_Bi_0.4_**+G, 2D **Sb_0.6_Bi_0.4_**, and HD-**Sb_0.6_Bi_0.4_**@G electrodes.


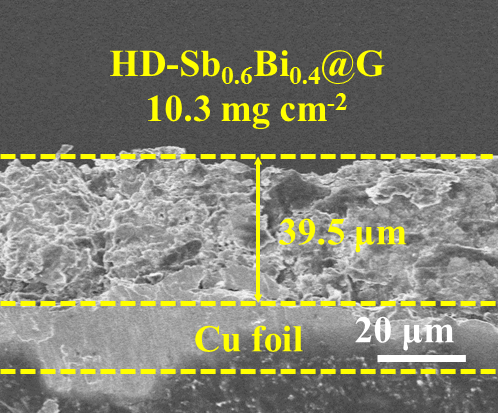


**Figure S8**. The relationship between thickness and mass loading of the HD-Sb_0.6_Bi_0.4_@G electrode.

**
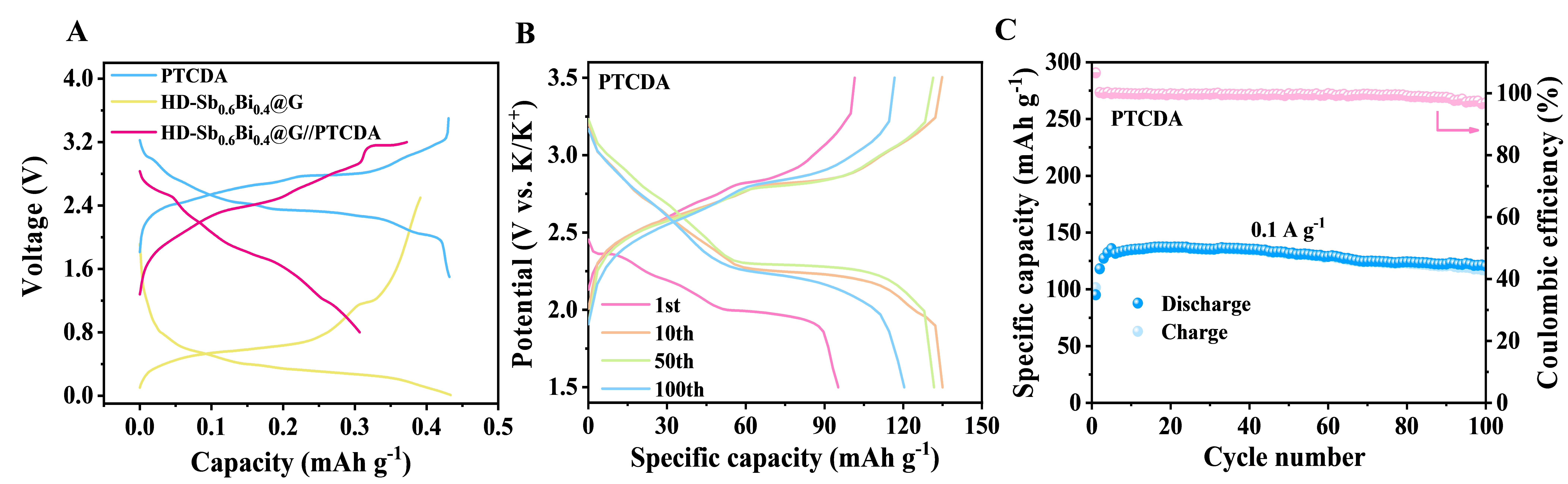
**

**Figure S9**. (A) GCD curves of HD-Sb_0.6_Bi_0.4_@G andode, PTCDA cathode and HD-Sb_0.6_Bi_0.4_@G //PTCDA full cell at 0.2 A g^-1^. (B) Galvanostatic charge-discharge (GCD) profiles of the PTCDA cathode at 0.1 A g^-1^. (C) Cycling performance of the PTCDA cathode at 0.1 A g^-1^.


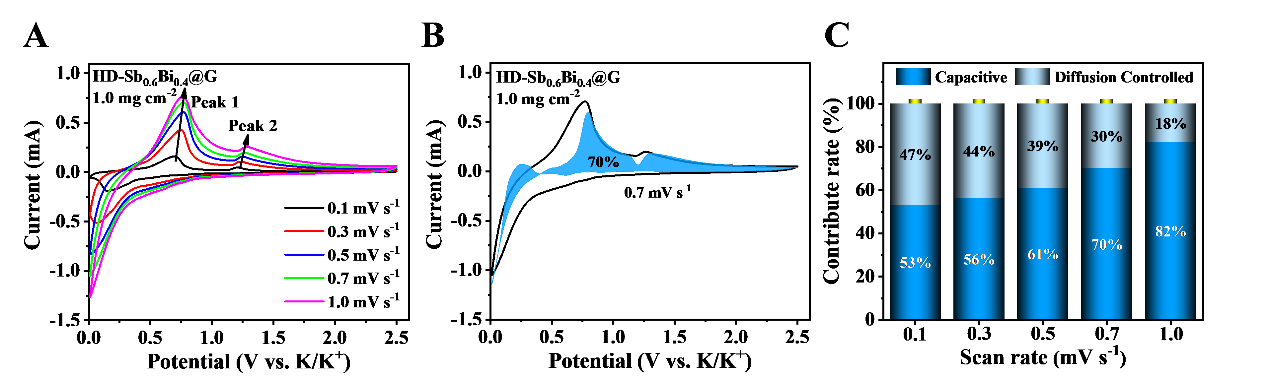


**Figure S10**. (A) The CV curves of the HD-Sb_0.6_Bi_0.4_@G electrode at a loading mass of 1.0 mg cm^-2^ under scan rates ranging from 0.1 to 1 mV s^-1^. (B) Its capacitive-controlled contributions at 0.7 mV s^-1^. (C) Normalized ratios of capacitive-controlled contribution at various scan rates.


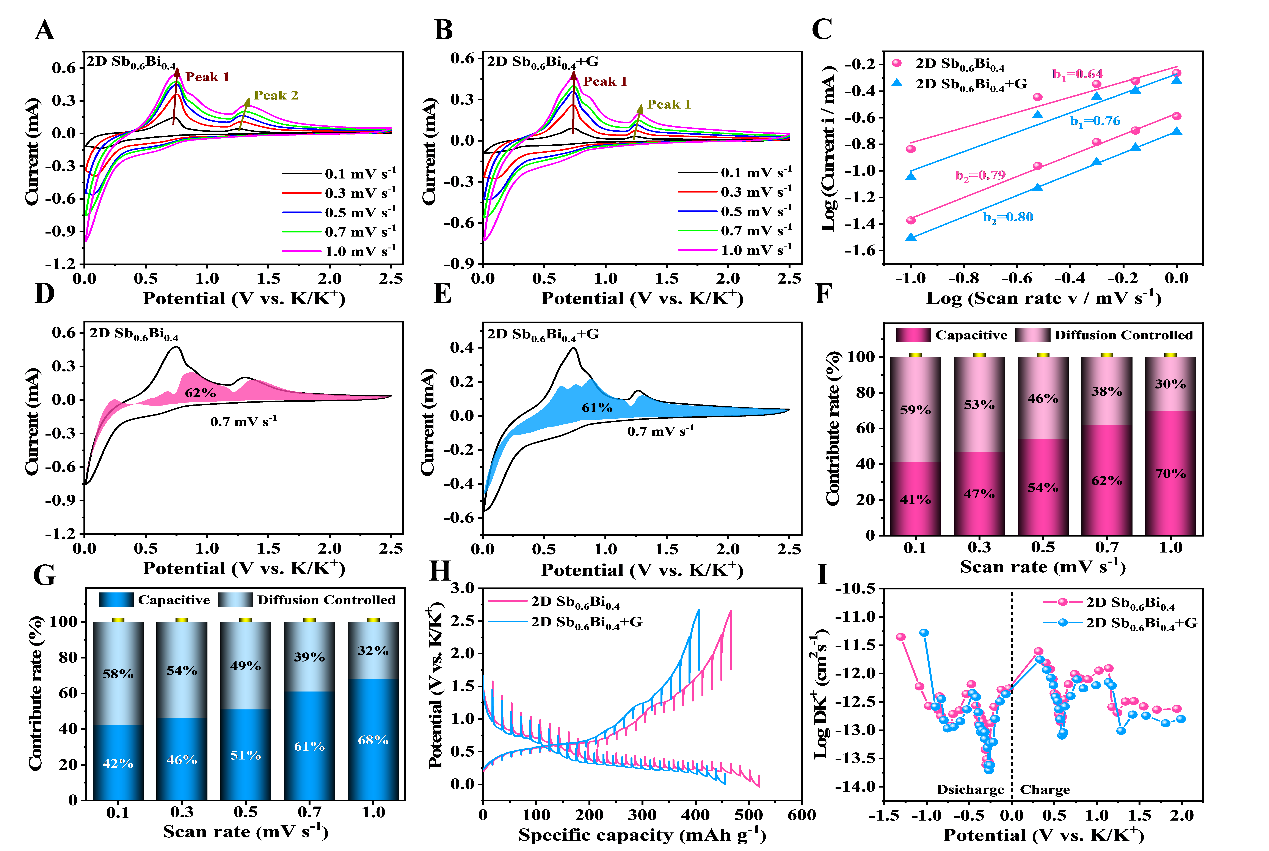


**Figure S11**. (A, B) CV curves at the loading of 1 mg cm^-2^ at the scan rates of 0.1 to 1 mV s^-1^. (C) relationship between log (i) vs. log (v). (D, E) capacitive and diffusion-controlled contributions at 0.7 mV s^-1^. (F, G) normalized ratios of capacitive-controlled contribution at various scan rates. (H) GITT curves and (I) diffusion coefficient of K^+^ during discharge/charge proces.

.
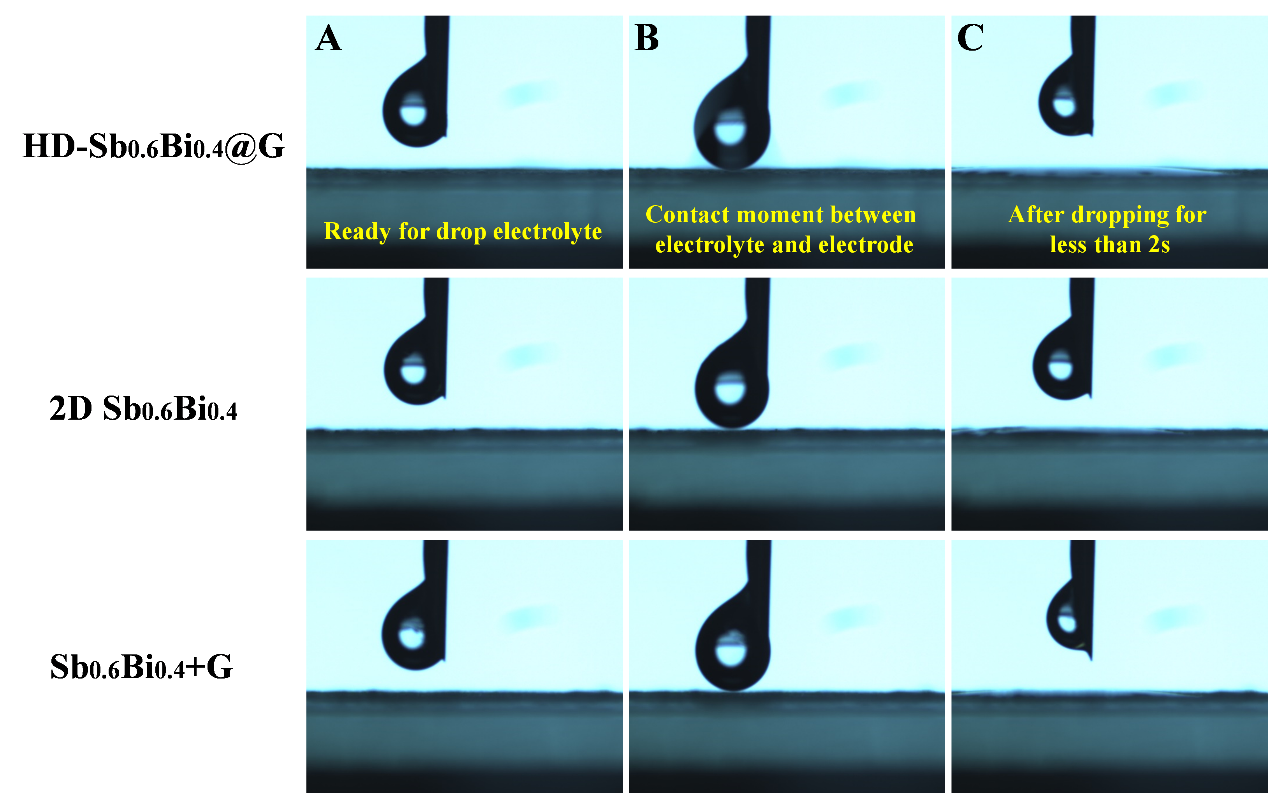


**Figure S12**. (A-C) Contact angle experiments of HD-Sb_0.6_Bi_0.4_@G electrode with electrolyte.


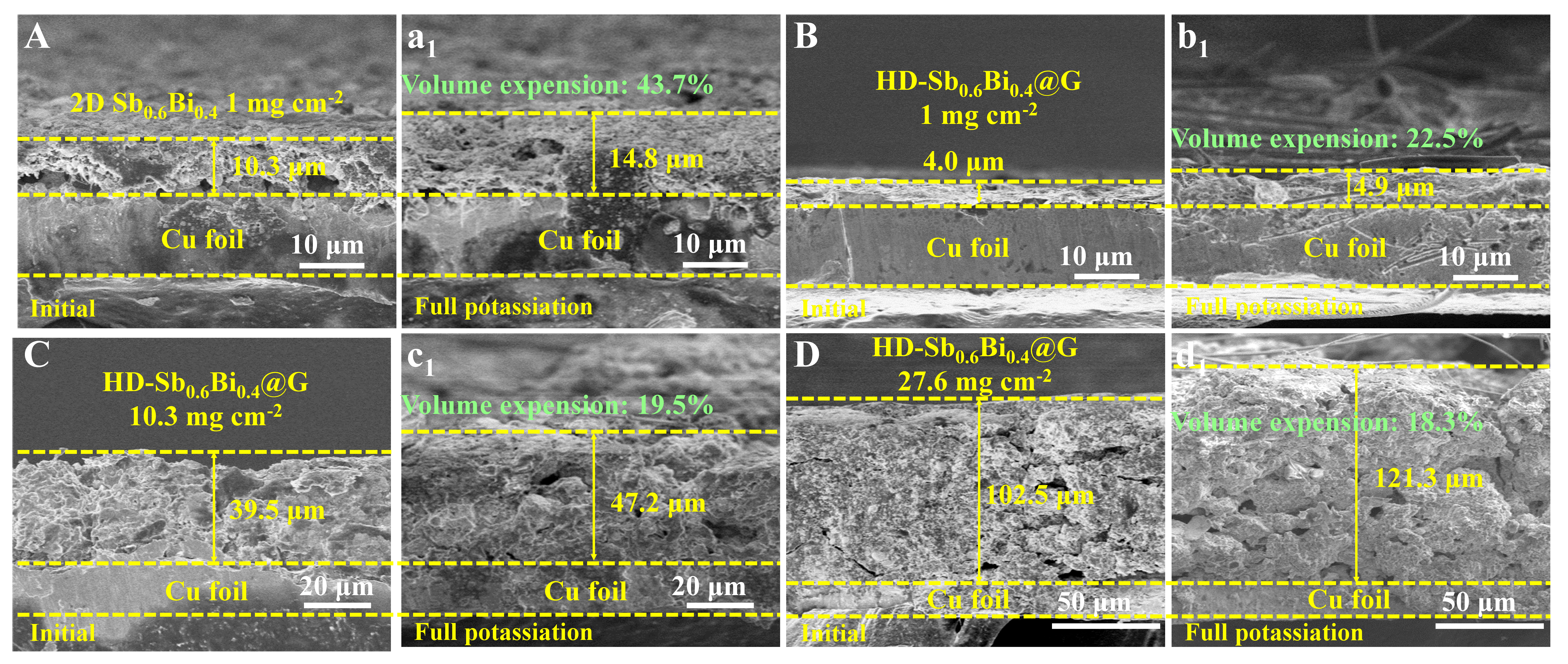


**Figure S13**. (A) Changes of electrode thickness before and after potassiation of 2D Sb_0.6_Bi_0.4_ anode at 1.0 mg cm^-2^ loading and (B) HD-Sb_0.6_Bi_0.4_@G at loading of 1.0 mg cm^-2^, (C) 10.3 mg cm^-2^ and (D) 27.6 mg cm^-2^.


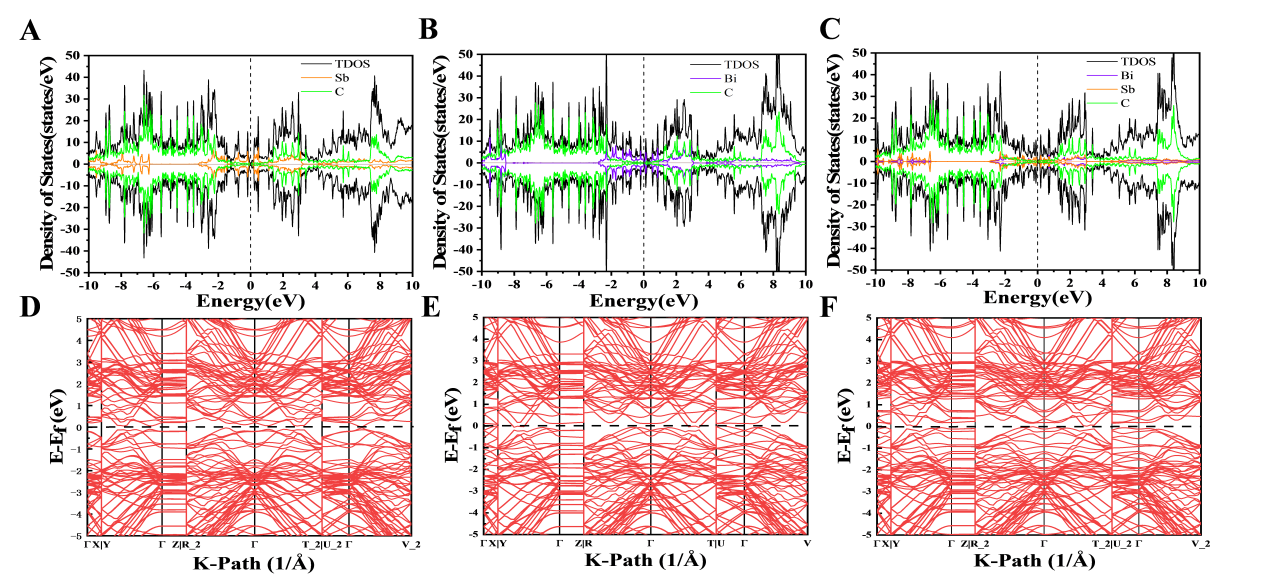


**Figure S14. Comparison of (A-C) band structures and (D-E) density of states (DOS) for K^+^ surface diffusion on HD-Sb@G, HD-Bi@G, and HD-Sb_0.6_Bi_0.4_@G based on DFT calculations.**

**Table S1.** Electrochemical performance comparison of areal/volumetric capacity of HD-Sb_0.6_Bi_0.4_@G monolith anode with most of reported representative anodes for PIBs.

| **Materials** | **Content of active material**  **in electrode (%)** | **^a^Max (gravimetric)/volumetric capacity based on the whole electrode**  **(mAh cm^-3^)** | **Highest areal capacity**  **(mAh cm^-2^)**  **^b^at max mass loading**  **(mg cm^-2^)** | **Electrode density**  **(g cm^-3^)** | **Refs.** |
| --- | --- | --- | --- | --- | --- |
| CNT/S-TiO_2_ | 90 | 196/157.4/0.1 A g^-1^  (at 1.96 mg cm^-2^) | 3.40  21.2 | 0.98 | ^[1]^ |
| CSSe@CNFs | 100 | 408.7/752/0.05 A g^-1^  (at 1.0 mg cm^-2^) | 7.58  25.3 | 1.84 | ^[2]^ |
| **CMPTO** | 92 | 333.5/1111.1/0.02 A g^-1^  (at 1 mg cm^-2^) | ~2  5.6 | ~1 | ^[3]^ |
| **CoTe_2_@NPCNFs@NC** | 75 | 513.1/1393.9/0.05 A g^-1^  (at 1.2 mg cm^-2^) | 0.62  1.2 | 2.72 | ^[4]^ |
| MoSSe | 80 | 376/854/0.05 A g^-1^  (at 1.0 mg cm^-2^) | 0.38  1.0 | 2.27 | ^[5]^ |
| Mn-PAA/FG | 80 | 1046/1203/0.1 A g^-1^  (at ~6.5 mg/cm^-2^) | 7.3  18.0 | 1.15 | ^[6]^ |
| Red P/SWCNTs | 80 | 1658/829/0.125 A g^-1^  (at 0.63 mg cm^-2^) | 10  10.74 | 1.07 | ^[7]^ |
| BFO-MF | 80 | 560/1120/0.05 A g^-1^  (at 1 mg cm^-2^) | 0.84  1.5 | 2.0 | ^[8]^ |
| **Bi@C-NSA** | 100 | 372.7/670.9/0.05 A g^-1^  (at 1 mg cm^-2^) | 0.932  2.5 | 1.8 | ^[9]^ |
| Bi_6_Sn_4_ | 70 | 584/1226/0.1 A g^-1^  (at 1.0 mg/cm^2^) | 0.8  1.3 | 2.1 | ^[10]^ |
| **HD-Sb_0.6_Bi_0.4_@G** | **90** | **521.2/1355.1/0.1 A g^-1^ (1.0 mg/cm^2^)** | **11.4**  **27.6** | **2.6** | **This work** |

^a^ The max volumetric (gravimetric) capacity of the electrode was calculated based on the total volume (mass) of the whole electrode (including binder and conductive additive) at the low mass loading.

^b^ All areal capacity is based on the whole electrode (including binder and conductive additive).

**References**

[1] C. Wang, Q. Yao, M. Wang, C. Zheng, et al., "Highly Conductive Hierarchical TiO_2_ Micro-Sheet Enables Thick Electrodes in Sodium Storage," *Advanced Functional Materials* 34 (2023): 2301996, <http://doi.org/10.1002/adfm.202301996>.

[2] S. Chen, J. Zhong, H. Deng, Q. Wei, et al., "Advanced Potassium-Ion Batteries with High Areal Capacity," *CCS Chemistry* 6 (2024): 1011-1023, <http://doi.org/10.31635/ccschem.023.202302895>.

[3] W. Yang, J. Huang, Q. Zheng, L. Chen, et al., "A Bandgap-Tuned Tetragonal Perovskite as Zero‐Strain Anode for Potassium‐Ion Batteries," *Angewandte Chemie International Edition* 63 (2024): e202412706, <http://doi.org/10.1002/anie.202412706>.

[4] Q. Li, J. Peng, W. Zhang, L. Wang, et al., "Manipulating the Polytellurides of Metallic Telluride for Ultra‐Stable Potassium‐Ion Storage: A Case Study of Carbon‐Confined CoTe_2_ Nanofibers," *Advanced Energy Materials* 13 (2023): 2300150, <http://doi.org/10.1002/aenm.202300150>.

[5] W. Feng, X. Wei, F. Cao, Y. Li, et al., "Defective MoSSe with Local-Expanded Structure for High-Rate Potassium Ion Battery," *Energy Storage Materials* 65 (2024): 103186, <http://doi.org/10.1016/j.ensm.2024.103186>.

[6] K. Yong, H. Fang, B. Wang, X. Qiu, et al., "Synergistic Structural Engineering of Tunnel‐Type Polyantimonic Acid Enables Dual-Boosted Volumetric and Areal Lithium Energy Storage," *Advanced Energy Materials* 12 (2022): 2200653, <http://doi.org/10.1002/aenm.202200653>.

[7] H. Kaur, B. Konkena, C. Gabbett, R. Smith, et al., "Amorphous 2D‐Nanoplatelets of Red Phosphorus Obtained by Liquid‐Phase Exfoliation Yield High Areal Capacity Na‐Ion Battery Anodes," *Advanced Energy Materials* 13 (2022): 2203013, <http://doi.org/10.1002/aenm.202203013>.

[8] J. Guo, L. Wang, A. Hu, J. Zhang, et al., "3D Micro-Flower Structured BiFeO_3_ Constructing High Energy Efficiency/Stability Potassium Ion Batteries Over Wide Temperature Range," *Advanced Functional Materials* 34 (2024): 2313300, <http://doi.org/10.1002/adfm.202313300>.

[9] Y. Wang, X. Xu, Y. Wu, F. Li, et al., "Facile Galvanic Replacement Construction of Bi@C Nanosheets Array as Binder‐Free Anodes for Superior Sodium‐Ion Batteries," *Advanced Energy Materials* 14 (2024): 2401833, <http://doi.org/10.1002/aenm.202401833>.

[10] C. Xu, S. Sun, X. Zhang, H. Zhang, et al., "Microsized Alloying Particles with Engineered Eutectic Phase Boundaries Enable Fast Charging and Durable Sodium Storage," *Energy Storage Materials* 74 (2025): 103906, <http://doi.org/10.1016/j.ensm.2024.103906>.
